# Ultrafast Optoacoustics Reveals Intricate 3D Anisotropic Elasticity in Nanocrystalline Membranes

**DOI:** 10.1002/advs.76472

**Published:** 2026-07-07

**Authors:** Shuchang Zhang, Yi He, Guojie Luo, Jie Huang, Pak San Yip, Haoyu Cui, Wanglinhan Zhang, Zijian Wang, Lin Ye, Yang Lu, Zhongqing Su, Yehai Li

**Affiliations:** ^1^ Department of Mechanical Engineering The Hong Kong Polytechnic University Kowloon Hong Kong SAR China; ^2^ Department of Mechanical Engineering The University of Hong Kong Hong Kong Hong Kong SAR China; ^3^ School of Aeronautics and Astronautics Sun Yat‐sen University Shenzhen Guangdong China; ^4^ The School of Automation and Intelligent Manufacturing Southern University of Science and Technology Shenzhen Guangdong China; ^5^ Materials Innovation Institute For Life Sciences and Energy (MILES) HKU‐SIRI Shenzhen Guangdong China

**Keywords:** 3D anisotropic elasticity, multi‐parameter inversion, nanocrystalline membranes, ultrafast optoacoustics, zero‐group‐velocity resonances

## Abstract

The mechanical performance of nanocrystalline membranes plays a critical role in determining the reliability and stability of advanced integrated circuit devices and nano‐electromechanical systems. Conventional characterization techniques such as nano‐indentation and micro‐scale mechanical testing, while widely used, are generally destructive and incapable of resolving three‐dimensional (3D) anisotropic properties. Targeting nanocrystalline membranes, we introduce an ultrafast optoacoustics‐based approach for characterizing their 3D anisotropic elastic constants and thickness simultaneously. Gigahertz Lamb waves are thermoelastically generated to propagate in nanocrystalline copper membranes. Both non‐propagating zero‐group‐velocity resonances and propagating modes are captured in high spatial and temporal resolution. The resonance and dispersion characteristics are employed to determine the thickness and anisotropic elastic constants of the membrane via a nontrivial multi‐parameter inversion algorithm. Accordingly, substantially different elastic properties are observed by changing the substrate, especially the shear stiffness, which could be a result of the underlying microstructural mechanisms. The developed ultrafast optoacoustic approach provides a fully non‐destructive and in situ metrology tool for characterizing parameters such as 3D anisotropic elastic constants and thickness of freestanding membranes. This capability not only enables a deeper understanding of the mechanisms of nanocrystalline materials but also facilitates improvements in the design and fabrication of reliable, high‐performance next‐generation micro‐ and nano‐devices.

## Graphical abstract

Ultrafast optoacoustic spectroscopy combined with a Sagnac interferometer enables non‐contact, simultaneous characterization of thickness and the full three‐dimensional anisotropic elastic constants in freestanding nanocrystalline copper membranes. By capturing gigahertz Lamb waves and zero‐group‐velocity resonances followed by SAFE‐GA inversion, the method provides a powerful new tool for ultrathin‐film metrology.

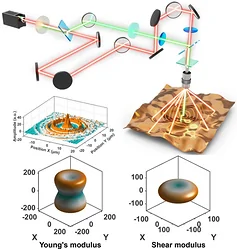

## 1 Introduction

Nanocrystalline membranes have emerged as a transformative class of materials with vast potential to redefine the boundaries of third‐generation semiconductors and advanced nanodevices. Meticulously engineered at the nanoscale with tailored crystallinity and microstructural architectures, these membranes are increasingly recognized as foundational components for post‐Moore electronics [1, 2, 3], advanced battery technologies [4, 5, 6], and nano‐electromechanical systems [7, 8]. The combination of their unique mechanical flexibility, electronic tunability, and reduced dimensionality enables unprecedentedly compact device architectures and functionalities that are unattainable with conventional bulk counterparts. Advances in ultrathin nanocrystalline membranes are accompanied by increased complexity and inhomogeneity in grain size, crystallographic orientation, and defect density [9, 10, 11]. These features are commonly induced during fabrication processes [12, 13, 14], leading to interfacial stress mismatches, subsurface anomalies, and layer delamination, which critically impact device performance and reliability [12, 15, 16, 17]. Unlocking the vast potential of ultrathin nanocrystalline membranes requires precise multiphysical characterization—particularly of mechanical properties—to accurately quantify the inherent nanoscale complexities of these ultrathin membranes.

To address such an impending need, a diversity of existing metrology approaches has been developed. Representatively, (i) the scanning probe nanoscale methods, as typified by nanoindentation [18, 19] and atomic force microscopy [20, 21] (AFM), are capable of offering atomic‐scale spatial resolution. Nevertheless, their reliance on physical contact limits penetration depth and confines measurements typically to the surface and near‐surface features. Moreover, the delicate nature of ultrathin nanocrystalline membranes in a freestanding configuration makes them highly susceptible to damage, contamination, or artifacts during contact measurement, hindering accurate and reliable characterization of intrinsic mechanical properties. Furthermore, these techniques are used mostly to measure the elastic modulus along the thickness direction, failing to capture the 3D anisotropic mechanical response of ultrathin membranes, which can vary significantly with different nanocrystalline structures. (ii) Micromechanical methods, including micro‐tensile testing [22], bulge testing [23], and micro‐cantilever‐based approaches [24], offer certain advantages for probing the mechanical properties of thin membranes, such as direct access to stress–strain behavior, applicability to freestanding structures, and high sensitivity to in‐plane properties. However, these techniques typically require elaborate sample preparation procedures and delicate measurement fixtures, such as the fabrication of suspended structures or the attachment of micro‐manipulators, which can introduce additional sources of error or damage to the membranes under testing. Moreover, micro‐mechanical tests are typically performed ex situ, which limits their ability to monitor dynamic changes during membrane fabrication or device operation. Furthermore, similar to the scanning probe techniques, these methods measure the elastic modulus only in a single direction and cannot directly determine membrane thickness. (iii) Photonic and electronic methods, including x‐ray reflectivity (XRR) [25], X‐ray diffraction (XRD) [26], and ellipsometry [27], provide powerful non‐contact tools for characterizing certain structural and optical properties of ultrathin membranes, such as thickness, density, crystallinity, and refractive index. Electron microscopy techniques like transmission electron microscopy [28] (TEM) and scanning electron microscopy [29] (SEM) offer complementary high‐resolution characterization of morphology, with TEM enabling ultrahigh‐resolution imaging of internal nanostructures and thickness, and SEM providing detailed surface morphology and cross‐sectional information. Notwithstanding, these techniques are limited in their ability to directly probe mechanical properties such as elastic moduli, adhesion, or interfacial stress. In conclusion, despite advancements in current metrology techniques, there remains an urgent need for a non‐destructive, in situ, and comprehensive characterization method for ultrathin nanocrystalline membranes in both industrial and research settings.

On the other hand, ultrafast optoacoustic technology [30, 31, 32, 33, 34, 35, 36, 37, 38, 39, 40, 41, 42, 43] has emerged as a powerful candidate for characterizing ultrathin membranes. By harnessing ultrashort laser pulses, especially in the femtosecond to picosecond regime, high‐frequency coherent acoustic phonons can be generated and detected in the membranes, which can be employed for high‐resolution mechanical characterization. Notably, Xie [30] et al. use a femtosecond laser to investigate a bilayer consisting of an 1830 nm silicon‐nitride plate coated with a 660 nm titanium film. They successfully image the acoustic field and clearly capture zero group velocity (ZGV) resonance at 1.69 GHz with an experimental wavenumber of approximately 11.055 µm^−^^1^. Thelen [31] et al. explore guided elastic waves and ZGV resonances in a 105 µm thick nanoporous silicon membrane (∼55% porosity, 37 nm pore radius) using a picosecond laser, which reveals a nearly isotropic elasticity perpendicular to the pore axes. The nearly isotropic results significantly deviate from those of the bulk silicon. Illienko [32] et al. characterize a buried periodic nanostructure beneath ∼400 nm thick optically opaque zirconium membranes using a femtosecond laser, and they achieve nanoscale resolution. Tomoda [33] et al. demonstrate high‐resolution acoustic profiling in transparent fused silica coated with 470 nm polycrystalline titanium films using time‐domain Brillouin scattering. This study achieves ∼600 nm depth resolution, measures a sound velocity of 5.92 km/s at 27.5 GHz Brillouin frequency, and realizes a 3D volumetric mapping up to 20–25 µm depth. Lomonosov [34] et al. apply picosecond ultrasonics to ∼800 nm thick transparent nanoporous membranes, which demonstrates significant depth‐dependent variations: acoustic velocity changed by up to 30% and refractive index by ∼6% across the film thickness. This study facilitates simultaneous thickness‐resolved mechanical and optical profiling in a single transparent nanostructured layer. That said, despite these significant advances in obtaining mechanical (acoustic) properties and morphology of ultrathin structures, such measurements have largely been performed separately—typically relying on prior knowledge of thickness or elastic modulus and assuming material isotropy.

In this paper, we reveal the 3D anisotropic elasticity of nanocrystalline copper membranes using ultrafast optoacoustics. This approach enables simultaneous, non‐destructive quantification of both the thickness of the ultrathin nanocrystalline membrane and its elastic properties. A femtosecond pump‐probe platform combined with a Sagnac interferometer is developed to implement the full wavefield imaging of GHz acoustic waves, as illustrated schematically in Figure 1a. Multi‐mode waves are observed, including non‐propagating ZGV resonance and propagating Lamb waves (symmetric and antisymmetric), as shown in Figure 1b,c. First, the local ZGV resonance spectroscopy is used to accurately identify the elastic symmetry, and then frequency–wavenumber dispersion relations are extracted with a spatiotemporal scan (B‐scan) of the wavefield. Based on the dispersion relation, we use the semi‐analytical finite element modeling coupled with a genetic algorithm (SAFE‐GA) to simultaneously determine the 3D anisotropic elastic constants and absolute membrane thickness. We validate the method through finite element simulations across different anisotropic conditions and experimentally apply it to copper membranes deposited on different substrates (i.e., amorphous silicon nitride and single‐crystal silicon, denoted by Cu^SiN^ and Cu^Si^, respectively). Notably, we detect GHz Lamb waves and ZGV resonance up to 13 GHz in a copper membrane approximately 600 nm thick. We complete multi‐parameter inversion of anisotropic elastic constants and thickness of nanocrystalline copper membranes. This enables us to reveal the significant influence on membrane elastic properties by changing deposition substrates. The transverse elastic isotropy of the Cu^Si^ and Cu^SiN^ membranes is conclusively demonstrated by the analysis of ZGV Lamb wave modes, which remains consistent regardless of the crystalline structure of the underlying substrates. This behavior aligns with the inherently disordered, random growth characteristic of physical vapor deposition (PVD) processes. Especially, the substantial increase of the shear stiffness (*C*_44_) has been observed on Cu^Si^ membranes, compared to Cu^SiN^ membranes under identical deposition conditions, which has not yet been reported, to the best of the authors’ knowledge.

**FIGURE 1:**
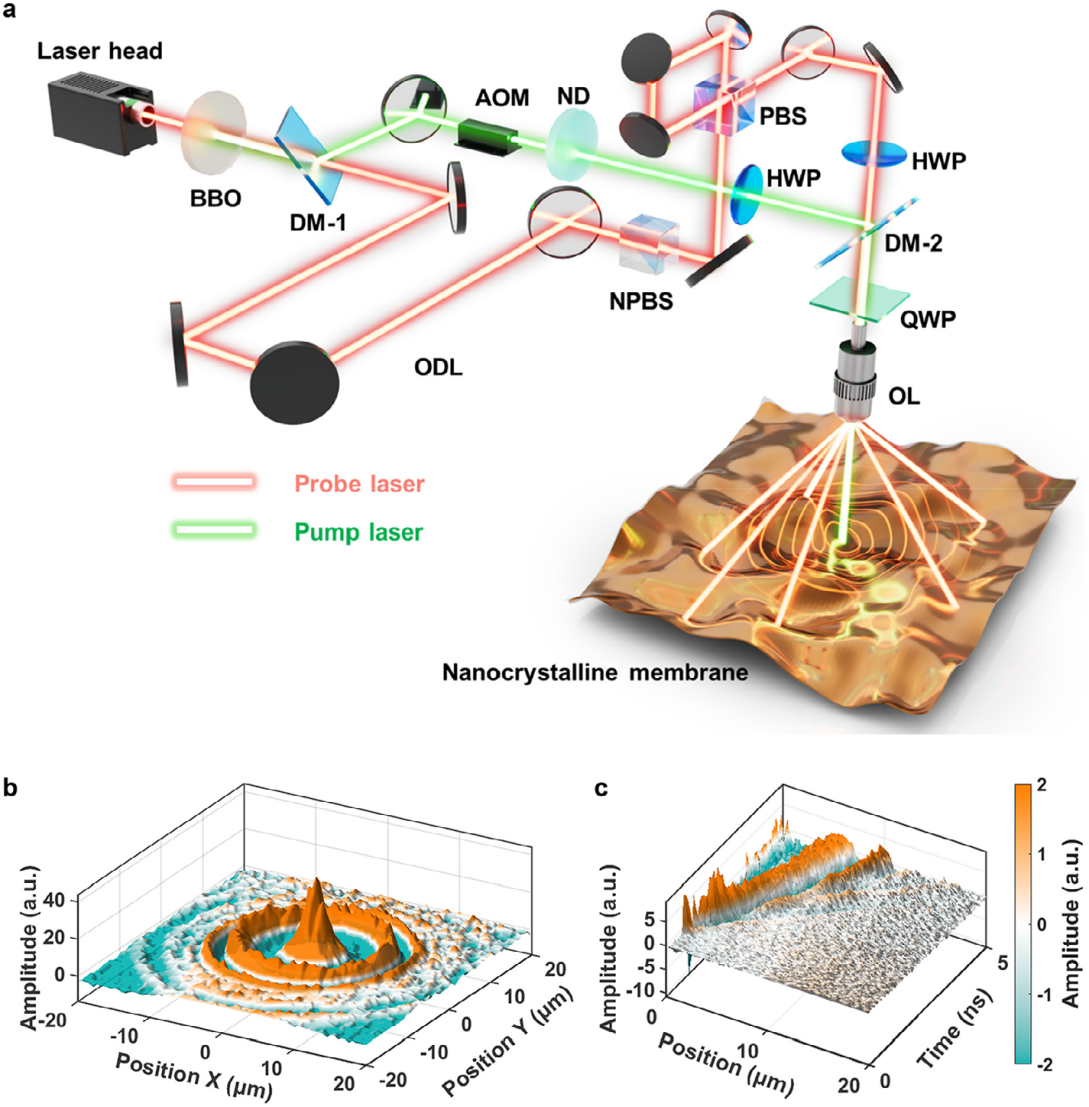
Principle of the proposed ultrafast optoacoustic characterization approach: (a) schematic of femtosecond pump‐probe platform with a Sagnac interferometer for GHz wavefield imaging (BBO: beta barium borate crystal, DM: dichroic mirror, AOM: acousto‐optic modulator, ODL: optical delay line, ND: neutral density filter, PBS: polarizing beam splitter, NPBS: non‐polarizing beam splitter, HWP: half wave plate, QWP: quarter wave plate, OL: objective lens, unlabeled mirrors are standard reflectors). (b) Wavefield map of Cu^Si^ membrane at 3 ns time delay. (c) Spatiotemporal wavefield of Cu^Si^ membrane.

## 2 Results

### 2.1 Freestanding Nanocrystalline Copper Membrane

Copper membranes are fabricated via electron‐beam (E‐beam) sputtering. They are deposited onto two types of substrates: silicon nitride (SiN) and single‐crystal silicon (Si (100)), each with an intermediate chromium barrier layer (Figure 2a). Freestanding membranes are released by plasma etching of the underlying SiN or Si substrate. Reactive ion etching (RIE) is employed, using a tailored gas mixture to selectively remove the substrate material beneath the copper membrane without damaging the membrane. Figure 2b–e present optical images of copper membranes under different processing stages. A uniform light‐yellow hue across the membrane window is observed for the copper film on a single‐crystal Si (100) substrate (Figure 2b). After the selective etching of the underlying silicon, the resulting freestanding Cu^Si^ membrane exhibits a light‐gray coloration (Figure 2c). The copper film deposited on an amorphous SiN substrate displays a characteristic deep blue color (Figure 2d). Finally, the released freestanding Cu^SiN^ membrane (Figure 2e) presents a yellow‐tinted area with faint contour fringes, suggesting a globally flat membrane under slight tension over millimeter‐scale dimensions.

**FIGURE 2:**
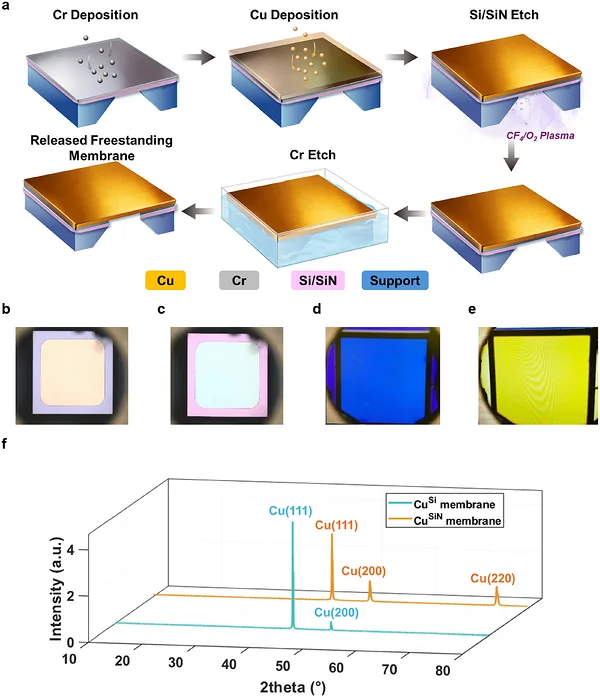
Nanocrystalline membrane fabrication process: (a) Schematic diagram of nanocrystalline copper membrane fabrication process (b) Cu on single‐crystal Si (100) membrane shows a uniform light‐yellow color across the membrane window. (c) Freestanding Cu^Si^ after substrate etching displays a light‐gray pattern. (d) Cu on amorphous SiN exhibits a deep blue color. (e) Freestanding Cu^SiN^ after substrate etching displays a yellow color. (f) XRD analysis of Cu^Si^ and Cu^SiN^ membranes.

X‐ray diffraction (XRD) characterization confirms the preferred crystal orientation of both types of membranes (Figure 2f). The Cu^SiN^ membrane displays multiple diffraction peaks corresponding to different crystal orientations, indicating a polycrystalline structure with random texture. In contrast, the Cu^Si^ membrane exhibits a pronounced (111) peak, which reveals the strong crystallographic texture. (For more details of the sample fabrication, see Section S4.1)

### 2.2 Anisotropy‐Driven Variations in ZGV

ZGV resonances are highly sensitive to the in‐plane elastic anisotropy of thin membranes. The resonance frequency exhibits measurable angular variation when the elastic properties differ with direction. To systematically investigate this anisotropy‐driven frequency variation, finite element method (FEM) simulations are performed using COMSOL Multiphysics with a fully coupled photo–thermal–elastic model to capture laser–driven transient heating, elastic–wave generation, and guided–wave propagation in freestanding copper membranes with prescribed microstructural symmetry. Three representative membranes are defined a priori: (i) single‐crystal Cu (111) membrane with cubic symmetry, (ii) single‐crystal Cu (100) membrane with cubic symmetry, and (iii) transversely isotropic Cu membrane. Symmetry identification is based on the first‐order ZGV resonance, a sharp and spatially localized spectral feature that exhibits high sensitivity to the elastic constants. Angle–resolved ZGV spectra were acquired by fixing the pump laser as a quasi–point source and rotating the probe laser azimuth *θ* with regard to the pump laser at a constant pump–probe offset of *r*_0_; the resulting angular distribution of the first–order ZGV frequency is used to identify the in–plane elastic symmetry and the principal axes. Figure 3 consolidates the identification of crystalline symmetry in copper membranes using the ZGV frequency method. The angle‐resolved fast Fourier transform (FFT) spectra for single‐crystal Cu (111) and Cu (100) membranes under different excitation offsets are displayed (Figure 3a,b). The corresponding polar plots of the normalized first‐order ZGV frequency reveal that, for the Cu (111) membrane, a distinct six‐fold (C_6_) symmetric pattern is observed at a 2 µm offset, which becomes less pronounced as the offset decreases (Figure 3c). Similarly, the Cu (100) membrane exhibits a clear four‐fold (C_4_) symmetry in its ZGV frequency distribution at both 1 and 2 µm offsets, with this signature diminishing at smaller offsets (Figure 3d). In contrast, the polar plot for the isotropic membrane manifests a perfect circular pattern at all offsets, confirming its in‐plane elastic isotropy (Figure 3e).

**FIGURE 3:**
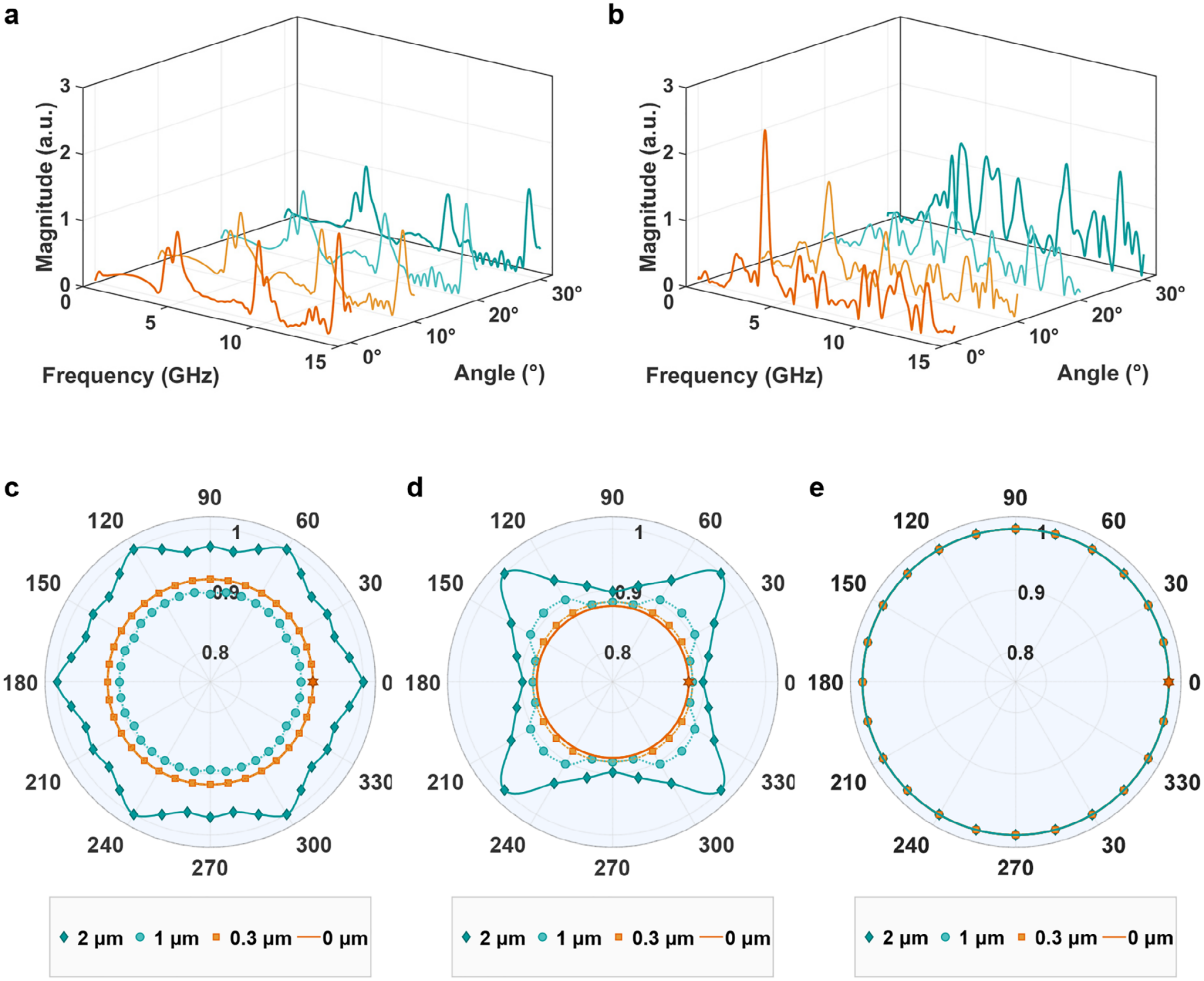
Simulated copper membrane symmetry identification by the ZGV frequency: (a) FFT spectra of time‐domain signals from single‐crystal Cu (111) membranes with offsets of 2 µm. (b) FFT spectra of time‐domain signals from single‐crystal Cu (100) membranes with offsets of 2 µm. (c–e) Polar plots of normalized ZGV frequencies comparing Cu (111) (c), Cu (100) (d), and isotropic Cu membrane (e) with different offsets.

The appropriate pump–probe offset *r*_0_ enhances the effectiveness of the measurement of angular‐sensitive ZGV. This enhancement occurs because the energy flux of the ZGV resonance in anisotropic materials is directionally dependent, leading to a non‐uniform spatial distribution of vibrational energy around the excitation point. At the origin (*r*_0_ ≈ 0), the probe laser averages the sub‐wavelength acoustic field over its spot size. In this region, contributions from all azimuthal directions overlap coherently, which leads to an averaging of the direction‐dependent ZGV resonance frequencies and thereby reduces angular sensitivity. Introducing a finite offset allows the energy emitted by the point‐like pump laser to undergo short‐range propagation before the detection of the ZGV waves. Over this distance, the anisotropy of the material generates a nonzero transverse group‐velocity component that steers the energy flux away from the wave vector along those non‐principal directions [44], which deflects the energy flux away from the wave‐vector direction. Consequently, as the pump–probe offset increases, directional selectivity of the ZGV resonance improves, which allows the probe laser to predominantly capture contributions from directions aligned closer to the wave vector. In simulations on a 550 nm single‐crystal copper membrane, at small offsets (e.g., *r*_0_ = 0.3 µm for a 550 nm Cu membrane), the probe laser remains in the sub‐wavelength mixing regime, and the extracted ZGV frequency is essentially equal to the center (on‐axis) frequency, with negligible angular modulation. Similarly, the probe laser remains within the sub‐wavelength mixing regime when *r*_0_ = 1 µm, so only a small systematic ZGV frequency shift and weak angular modulation can be observed. On the other hand, at *r*_0_ = 2 µm (approximately equal to the wavelength of ZGV under these conditions), energy‐flow steering separates azimuthal components sufficiently to yield robust, azimuth‐dependent ZGV peak shifts that track the principal axes while preserving strong local resonance amplitude. Provided the offset is further increased to 3 µm, comparable angular shifts persist (anisotropy confirmed), but the ZGV amplitude is markedly diminished due to multipath interference over the longer propagation. Consequently, pump–probe offsets approximately equal to one guided‐wave wavelength preserve the spatial localization of the ZGV resonance while leveraging anisotropic energy‐flux steering to maximize angular contrast. Whereas offsets being out of such a range overly attenuate and smear the ZGV frequency shift. At a given frequency and thickness, the optimal operating window is centered at near *r*_0_ ≈ 2 µm, which strikes a balance between the directional separation and signal strength, and thus it enables reliable identification of the principal axes. At smaller offsets, the sub‐wavelength mixing degrades angular resolution, whereas at larger offsets, the ZGV resonance becomes weak to guarantee accurate detection.

### 2.3 Anisotropic Elasticity of Nanocrystalline Membrane

Building on the high angular sensitivity of the first‐order ZGV resonance and the optimal pump–probe offset established through FEM simulations, we apply the principal‐axis identification method to the freestanding nanocrystalline membranes. This approach is implemented on the Cu^SiN^ and Cu^Si^ membranes, each ∼550 nm thick and deposited on silicon nitride and single‐crystal Si (100) substrates, respectively. For each sample, transient out‐of‐plane velocity is recorded while rotating the probe laser azimuth at a fixed offset of 2 µm—a geometry chosen to suppress sub‐wavelength mixing and to resolve direction‐dependent wave components. Symmetry is identified by probing locally exciting the first‐order ZGV resonance over multiple in‐plane angles (Figure 4a). Although the two membranes exhibit nonidentical angular patterns, both ZGV polar patterns are nearly circular without preferred axes, indicating transverse isotropy in the membrane plane (Figure 4b). Despite this shared in‐plane symmetry, the two membranes show different absolute first‐order ZGV frequencies under identical measurement conditions. Although XRD reveals a strong out‐of‐plane Cu(111) texture in the Cu^Si^ membrane, the nanocrystalline copper films are not perfect single crystals. Our cross‐sectional TEM characterization confirms a dominant (111) texture along the film normal, together with a moderate density of coherent nanotwins. In addition, the plan‐view SEM image shows equiaxed grains in the film plane (for original SEM and TEM results, see Figures S13 and S14 in Section S4.3). This microstructure produces a strong crystallographic preference along the out‐of‐plane direction while the in‐plane grain orientations remain largely random, resulting in macroscopic transverse isotropy of the freestanding membrane. Consequently, the observed nearly circular ZGV polar plots reflect this transverse isotropy, as the ZGV resonance frequency is insensitive to the azimuthal angle in the film plane. Achieving a clear six‐fold symmetry in the ZGV polar plots would require significantly higher crystallographic purity and near‐perfect in‐plane ordering, which is not the case here. The angular invariance of the ZGV frequency therefore establishes the transverse isotropy of the membrane plane, which in turn determines the appropriate form of the elastic stiffness tensor (with only five independent constants) for subsequent modeling. Accordingly, the angular invariance of the ZGV frequency establishes the transverse isotropy of the membrane plane, thereby fixing the appropriate form of the elastic stiffness tensor (with only five independent constants) for subsequent modeling. With this, we adopt a SAFE‐GA‐based inversion in conjunction with the use of a semi‐analytical finite‐element plate model to quantitatively extract elastic parameters.

**FIGURE 4:**
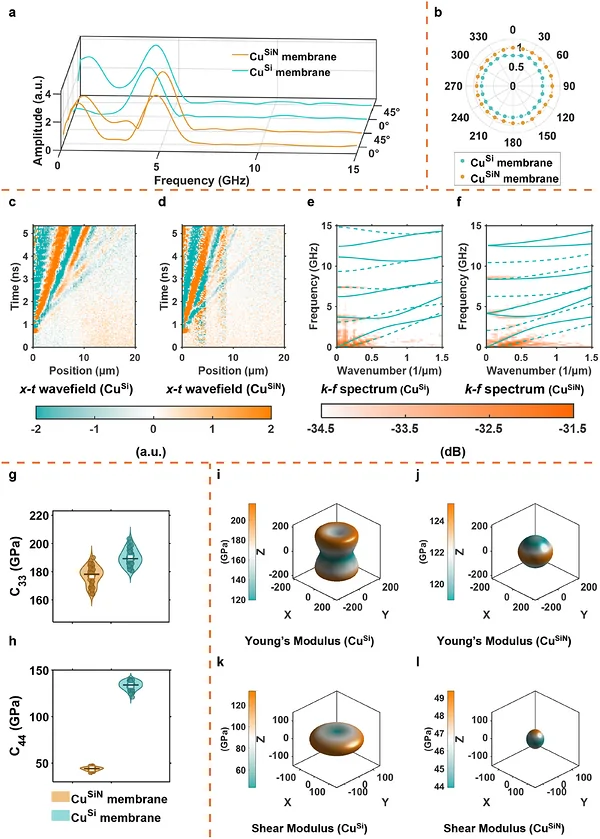
Experimental results obtained from Cu^Si^ membrane and Cu^SiN^ membrane: (a) FFT spectra of time‐domain signals from the two membranes with offsets of 2 µm. (b) Polar plots of normalized ZGV frequencies comparing the two membranes. (c) Spatiotemporal wavefield of Cu^Si^ membrane. (d) Spatiotemporal wavefield of Cu^SiN^ membrane. (e) Wavenumber–frequency spectrum of Cu^Si^ membrane with the inverted dispersion curves. (f) Wavenumber–frequency spectrum of Cu^SiN^ membrane with the inverted dispersion curves. (g) Retrieved *C*_33_ from SAFE‐GA inversion. (h) Retrieved *C*_44_ from SAFE‐GA inversion. (i) Three‐dimensional polar representation of equivalent Young's modulus for Cu^Si^ membrane. (j) Three‐dimensional polar representation of equivalent Young's modulus for Cu^SiN^ membrane. (k) Three‐dimensional polar representation of equivalent shear modulus for Cu^Si^ membrane. (l) Three‐dimensional polar representation of equivalent shear modulus for Cu^SiN^ membrane.

Following the principal axis determination method above, the SAFE‐GA algorithm is used to inversely solve the full elastic stiffness of these two nanocrystalline membranes. The ultrafast optoacoustic spatiotemporal scan is conducted first. The spatiotemporal (*x–t*) wavefield obtained from the two membranes both shows multiple well‐resolved fronts across ∼20 µm, which facilitates reliable dispersion extraction (Figure 4c,d). Subsequently, the wavenumber–frequency (*k–f*) intensity map was extracted from the reconstructed wavefield and fitted to theoretical dispersion curves via the SAFE‐GA algorithm, thereby allowing retrieval of the elastic stiffness (Figure 4e,f). For the Cu^SiN^ membrane, the distributions of SAFE‐GA results are tight and centered near *C*_11_ =  175.22 GPa, *C*_13_  =  84.75 GPa, *C*_33_  =  177.08 GPa, *C*_44_  =  43.93 GPa, and *h*  =  529.77 nm. For a transversely isotropic material, the degree of elastic anisotropy is quantified using the Thomsen anisotropy parameters ε and δ, which are defined as $\varepsilon =\frac{C_{11}-C_{33}}{2C_{33}}$ and $\delta =\frac{{\left(C_{13}+C_{44}\right)}^{2}-{\left(C_{33}-C_{44}\right)}^{2}}{2C_{33}\left(C_{33}-C_{44}\right)}$ [45]. These parameters measure the deviation of the in‐plane longitudinal modulus from the out‐of‐plane modulus of the Lamb waves, respectively. In general, values closer to zero indicate weaker anisotropy and a closer approach to isotropy. For the Cu^SiN^ membrane, the calculated values are ε  =   − 0.0053 and δ  =   − 0.0248, which reveals extremely weak anisotropy that is nearly isotropic. On the other hand, the SAFE–GA results converge on *C*_11_ =  166.74 GPa, *C*_13_  =  74.06 GPa, *C*_33_  =  189.92 GPa, *C*_44_  =  133.58 GPa, and *h*  =  623.12 nm for the Cu^Si^ membrane. Compared to the Cu^SiN^ membrane, increased elastic coefficients *C*_33_ and *C*_44_ can be observed for the Cu^Si^ membrane (Figure 4g,h). Remarkably, the value of C_44_ is almost threefold higher, which results in a substantial enhancement of the anisotropy coefficient (ε  =   − 0.0610 and δ  =  1.8663).

Spatially resolved 3D reconstructions of the elastic moduli (Figure 4i–l) further illustrate this contrast. The 3D polar plots were generated directly from the inverted stiffness tensors using the following expressions (no normalization; actual modulus values in GPa are shown):

For the equivalent (directional) Young's modulus *E*(θ) [46, 47]:

$$E\left(\theta \right)=\frac{1}{S_{11}\mathrm{si}n^{4}\theta +S_{33}\mathrm{co}s^{4}\theta +\left(2S_{13}+S_{44}\right)\mathrm{si}n^{2}\theta \mathrm{co}s^{2}\theta }$$

For the directional shear modulus *G*(θ):

$$G\left(\theta \right)=\frac{1}{S_{44}\mathrm{si}n^{2}\theta +S_{66}\mathrm{co}s^{2}\theta }$$

where θ is the polar angle from the out‐of‐plane *z*‐axis (symmetry axis), *S_ij_* are the compliance constants obtained by inverting the stiffness tensor, and *C*_66_ = (*C*_11_ − *C*_12_) /2 due to transverse isotropy. The plots are independent of the azimuthal angle. Note that because *C*_12_ exhibits low sensitivity under the current out‐of‐plane detection configuration and cannot be reliably inverted; its value in the directional shear modulus calculation was fixed at the ensemble‐averaged result from the 50 independent GA runs (in the range of 75–80 GPa), which lies well within the commonly reported range for copper.

The Cu^Si^ membrane exhibits pronounced out‐of‐plane anisotropy in both Young's modulus (∼120–200 GPa, dumbbell‐like pattern) and shear modulus (∼60–120 GPa, lobed features), whereas the Cu^SiN^ membrane displays nearly isotropic, uniform distributions (Young's ∼120–124 GPa; shear ∼44–49 GPa, near‐spherical), confirming the distinct effects of Si vs. SiN substrates on elastic anisotropy in the nanocrystalline copper deposition. Profilometry confirms thicknesses of 519.5 nm (Cu^SiN^) and 604.7 nm (Cu^Si^), agreeing closely with SAFE–GA posterior means of 529.77 nm (relative error ≈ 1.9%) and 623.12 nm (relative error ≈ 3.1%) (For original XRD and profilometry results, see Figures S7 and S8 in Section S4.2.3), respectively, recovered from ultrafast optoacoustic data—demonstrating robust sub‐micron thickness determination in nanocrystalline membranes. (For original time domain signals and inversion results, see Figures S11 and S12 in Section S4.2.3).

XRD results indicate that the Cu^SiN^ membrane exhibits a polycrystalline structure with multiple diffraction peaks, consistent with the transverse isotropy revealed by ZGV characterization. In contrast, the Cu^Si^ membrane displays a strong (111) preferred orientation. It should be noted that, although XRD indicates a pronounced (111) texture in the Cu^Si^ membrane, the angular ZGV polar plots remain nearly circular, demonstrating transverse isotropy rather than the C_6_ hexagonal symmetry expected for a pure (111) single‐crystal. This observation is consistent with the random nature of films grown by PVD (for original FFT spectra of ZGV signals, see Figure S10 in Section S4.2.3). The significantly elevated *C*_44_ value obtained from the SAFE–GA inversion substantially exceeds the shear modulus of single‐crystal copper. Our TEM characterization performed on the Cu film deposited under identical conditions directly reveals a strong and predominant (111) out‐of‐plane texture together with coherent nanotwins present at moderate density. Besides, molecular dynamics simulations have demonstrated that FCC/HCP coexistence occurs in Cu thin films deposited on single‐crystal (100)Si [9], particularly near substrates such as Si. Concurrently, EBSD and TEM characterizations of similar (111)‐textured Cu films frequently reveal dense nanotwin networks [48, 49, 50], which are known to enhance macroscopic shear resistance through interface‐mediated dislocation impediment. Based on these observations, we hypothesize that the increase in *C*_33_, together with the remarkably high *C*_44_ in the Cu^Si^ membrane, may arise from the strong (111) out‐of‐plane texture combined with the moderate density of coherent nanotwins observed in our TEM characterization. At the same time, other mechanisms—such as possible metastable FCC/HCP phase coexistence or additional defect structures—cannot be ruled out and may also contribute to the elevated stiffness [51, 52, 53]. (for original TEM results, see Figures S13 and S14 in Section S4.3)

## 3 Discussion and Conclusions

The combined FEM simulations and ultrafast optoacoustic experiments show that ZGV‐based spectral analysis effectively evaluates in‐plane symmetry and serves as a robust precursor for quantitative anisotropic elastic inversion. In single‐crystal copper membranes, the strong angular contrast of the first ZGV mode directly maps the principal axes. This anisotropy arises from tight coupling between ZGV strain localization and the stiffness tensor, yielding persistent frequency markers even amid mixed or weak propagating modes. However, such angular contrast of ZGV frequency shift cannot be properly observed until a moderate pump–probe offset is introduced. In contrast, transversely isotropic membranes exhibit rotational invariance in ZGV frequencies, producing near‐circular polar plots across thicknesses and confirming the absence of directional modulation. This clear distinction highlights the sensitivity of ZGV resonance to in‐plane symmetry classification and its reliability as an orientation marker. Experimental ZGV analysis of freestanding Cu^Si^ and Cu^SiN^ membranes shows consistent angular invariance, classifying both as transversely isotropic in the in‐plane direction. Although in‐plane symmetry exhibits similarity across deposition substrates, systematic ZGV frequency shifts revealed elastic differences driven by the different crystal morphology of the substrates. Thus, ZGV resonances can act simultaneously as in‐plane symmetry indicators and quantitative anchors for elastic constants, providing sufficient constraints to reduce parameters in anisotropic inversion models.

A SAFE–GA inversion algorithm is developed based on dispersion features extracted from frequency–wavenumber spectra. These spectra are obtained through spatiotemporal imaging of Lamb waves and incorporate prior ZGV resonance analysis to determine the elastic symmetry of the membranes. In simulations of single‐crystal copper membranes and transversely isotropic copper membranes, dispersion of Lamb waves reconstructs the 3D elastic constants and thickness with accuracy better than 5%. Low‐frequency modes primarily constrained shear and in‐plane longitudinal stiffness, while higher‐order cutoffs refined thickness and coupled stiffness. Integration of dispersion mapping with prior ZGV symmetry identification enabled stable and precise multi‐parameter inversion despite mode crossings and incomplete spectral coverage. In experiments, such an inversion algorithm successfully distinguishes Cu^Si^ and Cu^SiN^ membranes despite their similar in‐plane elastic symmetry. Elastic characterization shows pronounced transverse isotropy in the Cu^Si^ membrane, arising from random in‐plane distribution of phase boundaries and grain orientations. The elevated *C*_33_ correlates with the strong (111) texture observed by XRD. The significantly enhanced *C*_44_, which substantially exceeds the shear modulus of single‐crystal copper, may result from the synergistic contributions of (i) the dominant (111) orientation, (ii) the metastable FCC/HCP dual‐phase structure, and (iii) the presence of coherent nanotwins. These microstructural features collectively constrain dislocation‐mediated shear deformation in a composite‐like manner.

The observed substrate‐dependent elastic properties, even after the membranes are released to a freestanding state, can be understood through the memory effect of the substrate during the PVD process. During electron‐beam sputtering, the initial nucleation and growth of Cu atoms are strongly templated by the atomic arrangement of the underlying substrate. On the amorphous SiN substrate, the lack of long‐range order leads to random nucleation, resulting in a polycrystalline film with multiple crystallographic orientations and relatively weak texture (as confirmed by XRD). In contrast, the crystalline Si(100) substrate provides a well‐defined atomic template that favors nucleation of low‐surface‐energy (111) grains [9, 54, 55], which propagate through columnar growth to produce a strong (111) fiber texture together with a moderate density of coherent nanotwins (as directly confirmed by TEM in Figures S13 and S14). Importantly, the selective etching process used to release the membranes is chemically mild and does not alter the internal microstructure, crystallographic texture, or nanotwin distribution established during growth. Consequently, even after selective etching to obtain freestanding membranes, the Cu^Si^ membrane retains pronounced out‐of‐plane anisotropy, whereas the Cu^SiN^ membrane remains nearly isotropic. While these mechanisms have been widely explored in theory and simulations, their manifested macroscale elastic response has not been experimentally probed until this work. Further investigations are required to elucidate their respective contributions to 3D anisotropic elasticity.

The central innovation of this work is twofold. Methodologically, we demonstrate that ZGV resonances can be used not only to identify in‐plane symmetry but also to directly probe and confirm the form of the elastic matrix: the perfectly circular ZGV polar plots conclusively establish transverse isotropy of the freestanding nanocrystalline Cu membranes, thereby enabling reliable multi‐parameter inversion. In terms of application perspectives, we introduce the SAFE‐GA framework to achieve, for the first time, the simultaneous inversion of the 3D anisotropic elastic tensor together with absolute membrane thickness on truly ultrathin (∼600 nm) nanocrystalline copper membranes. Unlike previous ZGV‐based or ultrafast optoacoustic studies that typically assumed isotropy, known thickness, or simplified geometries, this combination of ZGV‐guided elastic‐matrix determination and SAFE‐GA‐based 3D‐tensor inversion allows us to reveal the pronounced substrate‐dependent out‐of‐plane anisotropy in real nanocrystalline films.

A key limitation of the current optical pump–probe approach is its reliance on strong thermoelastic excitation and high interferometric contrast, which are typically achieved in optically absorbing and reflective membranes. For transparent membranes, both the thermoelastic generation efficiency and the detection sensitivity are significantly reduced, which leads to weaker ZGV resonances and a lower signal‐to‐noise ratio in the dispersion branches. Mitigation strategies could involve ultrathin capping layers, immersion optics, or shorter‐wavelength probing. Additionally, the predominant use of out‐of‐plane velocity detection results in weak coupling to shear‐horizontal (SH) mode waves, leading to broader (less constrained) distributions of SAFE‐GA results for parameters such as *C*_12_ and *C*_13_. Polarization‐resolved detection or oblique‐incidence excitation schemes could enhance the sensitivity of detection to SH mode waves and improve the accuracy of these parameters. Finally, this study is limited to laboratory applications due to the complex optical path setup. Future improvements in optical setup and inversion algorithms could enable faster acquisition and broader wafer‐scale applications. Besides, the current mechanical scanning + optical delay line approach requires approximately several hours to characterize an entire wafer. In future work, this limitation can be overcome by replacing the optical delay line with asynchronous dual femtosecond lasers [56]. This asynchronous dual‐laser scheme enables rapid, continuous delay scanning without mechanical movement, potentially reducing the total measurement time for a full wafer to within several minutes — approaching the speed of conventional ultrasonic metrology — while maintaining the non‐contact and ambient‐condition advantages. Such improvement would make the technique highly suitable for in situ or wafer‐level metrology in industrial semiconductor fabrication lines.

In summary, this study characterizes the 3D anisotropic elastic properties and in‐plane symmetry in nanocrystalline ultrathin freestanding membranes using ultrafast optoacoustics. By combining high‐resolution ZGV resonance analysis with Lamb waves dispersion mapping in an integrated SAFE–GA inversion framework, the approach simultaneously determines the 3D anisotropic elastic constants and absolute membrane thickness with high accuracy, without prior assumptions about material symmetry or predefined thickness values. This method is employed to investigate substrate‐induced microstructural effects on the elastic properties of nanocrystalline copper membranes. Since the thickness is decoupled to consider fabrication variation, the anisotropy with multiple constants is successfully quantified, especially the high shear stiffness *C*_44_. This ultrafast optoacoustic method of fully non‐contact and non‐destructive nature exhibits the potential to be used as an in situ metrology technique for investigating evolution mechanisms and assessing structural properties of nano‐scale structures during fabrication or treatment. Extensions to other material systems are also under investigation, such as polymers or van der Waals heterostructures, which further elucidate the interfacial and anisotropic mechanics relevant to emerging flexible and quantum devices.

## 4 Methodology

### 4.1 Guided Wave Propagation and ZGV Phenomena in Thin Membranes

The acoustic characterization method is grounded in guided elastic wave theory—specifically, Lamb waves—in thin plates and membranes, where dispersion and propagation are controlled by the elastic stiffness tensor and crystal symmetry (isotropic, transversely isotropic, or cubic single crystal). In anisotropic media, direction‐dependent stiffness produces angular variations in phase and group velocities, polarization mixing, and mode coupling, which render guided waves highly sensitive to elastic anisotropy and microstructural texture. A key feature of Lamb wave dispersion is the presence of ZGV resonances [30, 44, 57, 58], defined by group velocity *v_g_ = ∂ω/∂k* = 0 while the phase velocity remains finite (*v_p_ = ω/k* ≠ 0). ZGV points are stationary extrema on dispersion curves and serve as sharp spectral markers of the stiffness tensor. Their frequencies shift systematically with anisotropy, thickness, and boundary conditions. This characteristic makes them reliable anchors for symmetry assessment and inversion, even when propagating content is weak or overlapping.

Principal elastic axes and the degree of in‐plane anisotropy are established using angular dispersion measurements of ZGV resonances derived from the ZGV frequency shift. ZGV resonances, measured as a function of in‐plane angle, exhibit characteristic angular modulation in anisotropic membranes, with frequency extrema directly indicating the principal elastic directions. In transversely isotropic membranes, by contrast, ZGV frequencies remain essentially angle‐invariant. This angular dependence (or invariance) of the ZGV frequencies provides a robust, spectrally anchored basis for determining principal axes and classifying symmetry before inversion. Forward modeling of guided wave dispersion calculation is carried out with a SAFE framework under Floquet–Bloch periodicity [59]. The SAFE method combines analytical periodicity along the propagation direction with finite‐element discretization through the thickness to form a tractable eigenvalue problem. The solver enforces traction‐free boundary conditions and incorporates anisotropic stiffness to compute direction‐dependent dispersion. (For further discussion of the SAFE method, see Figures S1 and S2 in Section S1.3).

### 4.2 SAFE‐GA Driven Parameter Inversion: Elastic Constants and Thickness

Quantitative extraction of elastic stiffness constants and membrane thickness from experimental or simulated *k‐f* data is performed using a hierarchical, physics‐constrained GA framework [60, 61]. The GA stochastically explores a physically admissible parameter space by initializing a population of chromosomes, each encoding a candidate set of elastic constants and thickness within stability‐enforced bounds. For every candidate, a forward dispersion solver—consistent with the SH mode wave missing—computes the corresponding *k‐f* relations, which are then compared against the experimental maps through a multi‐objective fitness that balances structural similarity, correlation, and amplitude agreement while penalizing proximity to elastic instability. The evolutionary loop employs rank‐based selection, constraint‐preserving blend crossover, and adaptive, sensitivity‐weighted mutation, with elite retention and diversity control to prevent premature convergence and a restart strategy to escape stagnation. To ensure statistical robustness, the GA is repeated over multiple independent runs; best solutions are aggregated after outlier rejection to yield means, standard deviations, and an averaged forward model, accompanied by final quality metrics and residual visualizations. This design tightly couples measurement physics and model constraints, enabling stable, high‐fidelity inversion for parameters most visible in out‐of‐plane motion, while acknowledging reduced sensitivity and broader posteriors for constants that rely on in‐plane polarization due to SH missing. Upon convergence, the optimal elastic constants and thickness are subjected to multivariate validation, including *k‐f* residual mapping, relative‐error quantification, and uncertainty propagation analysis, thereby linking the observed guided wave dynamics directly and rigorously to the underlying anisotropic elasticity of the membrane. (For further discussion of the SAFE‐GA inversion, see Figures S1–S3 in Section S1).

To validate the robustness of the inversion against noise, finite‐element simulations were performed using known true elastic constants for both isotropic and transversely isotropic cases, with realistic noise levels matching the experimental conditions. The SAFE‐GA algorithm recovered all six parameters with relative errors below 5% in both cases (see Figure S6 and Tables S1–S4 in Section S3). The robustness of the SAFE‐GA inversion is further demonstrated by a comprehensive statistical analysis of 50 independent GA runs and a detailed sensitivity analysis of the experimental results, including parameter distributions, pairwise correlation matrices, one‐dimensional fitness landscapes, and two‐dimensional slices of the multi‐objective fitness function (for the full statistical analysis and sensitivity analysis, see Tables S5–S8 and Figures S15–S18 in Section S5). These analyses confirm narrow parameter distributions with relative standard deviations typically below 6% for both membranes. The pairwise correlation matrices reveal the expected strong coupling between *C*_33_ and thickness (r = 0.938 for Cu^SiN^ and r = 0.965 for Cu^Si^). The sensitivity analysis further shows clear differences in parameter resolvability. *C*_33_, *C*_44_, and membrane thickness *h* are particularly sensitive, exhibiting sharp, prominent single peaks in the 1D fitness landscapes with large fitness variations on the vertical axis (Δfitness ≈ 0.01–0.055) and well‐defined, localized global minima in the 2D slices. *C*_11_ and *C*_13_ demonstrate moderate sensitivity, with smaller but visible fitness variations (Δfitness ≈ 0.002–0.004) and reasonably distinct minima in the corresponding 1D and 2D landscapes. In contrast, the fitness landscapes involving *C*_12_ are essentially flat in both 1D and 2D analyses, showing almost no variation with changes in *C*_12_ and confirming that this parameter cannot be reliably determined under the current out‐of‐plane detection scheme. Collectively, these results validate the stability and reliability of the multi‐parameter inversion. In particular, the main findings of this study — the significantly elevated *C*_33_ and especially the pronounced three‐fold increase in *C*_44_ for the Cu^Si^ membrane — are shown to be robust under the observed parameter coupling.

### 4.3 Photo‐Thermal‐Elastic FEM Simulation Modelling

FEM simulation is conducted using the COMSOL Multiphysics 6.1 platform, implementing a fully coupled photo‐thermal‐elastic model to capture the ultrafast optoacoustic response of copper membranes [62]. The simulation incorporates three interacting physical fields: (1) radiative beam absorption, where the laser pulse is modeled as a transient energy source and its absorption in the medium is governed by the Beer‐Lambert law; (2) heat transfer in solids, where the absorbed energy induces rapid temperature rise and transient thermal gradients, simulated using the heat conduction equation; and (3) solid mechanics (elasticity), where the resulting thermoelastic stresses drive elastic wave generation and propagation, solved via the elastodynamic equations. These fields are fully coupled, with radiative absorption providing the initial heat source for thermal conduction, which in turn generates stresses that act as input for the elastic wave simulation. The computational domain is discretized with a mesh size of 10–50 nm through the thickness to resolve GHz‐frequency acoustic waves, and time integration is performed using an implicit solver with adaptive time‐stepping for stability and accuracy. Boundary conditions are set as free surfaces at the membrane interfaces and periodic or absorbing boundaries laterally to minimize artificial reflections. Material anisotropy is explicitly encoded via the elastic tensor representation for each copper system. For single‐crystal copper, cubic symmetry is imposed, with three independent elastic constants. Transversely isotropic copper is modeled with five independent constants. The elastic tensors are implemented in the simulation environment according to the respective symmetry class, ensuring accurate representation of direction‐dependent stiffness and wave propagation characteristics.

To determine the principal elastic axes in anisotropic and transversely isotropic membranes, a three‐dimensional model is employed, computing ZGV resonances at propagation orientations sampled in 15° increments over the full 0°–360° range. A Gaussian‐shaped impulsive excitation (full‐width at half‐maximum 1 µm) is applied at the membrane center, and the resulting out‐of‐plane velocities at different offsets are recorded. The ZGV frequency is then extracted by applying a Fourier transform to each time trace and locating the dominant spectral peak near the predicted ZGV point. By plotting the ZGV frequency vs. orientation, the extrema—maxima and minima—are readily identified, and the corresponding angles are taken as the principal elastic axes for subsequent dispersion analysis.

Simulations of spatiotemporal scanning proceed along the selected principal axis with the pump laser fixed at the origin and the probe laser translated laterally in 0.2 µm increments over a 20 µm scan range. At each position, out‐of‐plane velocities are recorded over a 5 ns time window using 0.5 ps steps, assembled into a space–time matrix. A two‐dimensional FFT (with zero padding to double the resolution) transforms this into a frequency–wavenumber (*f–k*) map, revealing multiple guided Lamb‐wave branches. These maps serve as input to the SAFE–GA inversion. (For further discussion of the simulation modeling, see Figures S4 and S5 in Section S2).

### 4.4 Experimental Setup: Ultrafast Optoacoustic Measurement

Ultrafast optoacoustic measurements are performed using a femtosecond pump‐probe system designed for high‐sensitivity detection of acoustic waves, which is almost the same as the system in our previous study [59]. The pump laser, generated by a commercial laser source (wavelength: 1030 nm, pulse duration: ∼50 fs, repetition rate: 800 kHz), is focused onto the copper membrane surface to induce rapid local heating and generate broadband acoustic pulses via the thermoelastic effect. The pump spot size is typically ∼1 µm, and the incident energy is adjusted to avoid sample damage while ensuring sufficient acoustic excitation. This Sagnac interferometer introduces a relative optical path difference that is adjustable by fine‐tuning the positions of the three orthogonally placed reflectors. The center frequency of maximum sensitivity of the Sagnac interferometer is therefore tunable according to *f*  =  1/(2Δ*t*). In the present study, the optical path difference was adjusted to set the center frequency to approximately 5 GHz. In the present study, the optical path difference was adjusted to set the center frequency to approximately 5 GHz. The overall theoretical bandwidth of the system is bounded by two factors: the lowest measurable frequency of 417.78 MHz (determined by the maximum optical delay line range of 4.7873 ns) and the theoretical upper frequency limit of 1 THz (limited by the sampling rate). Typical acquisition time for a full B‐scan is about 2 h (no averaging). The laser fluence was kept at 0.3–0.5 mJ/cm^2^, well below the damage threshold of the Cu membranes.

ZGV spectroscopy is performed by fixing the femtosecond pump laser at a single point on the freestanding copper membrane and positioning the probe beam at a constant radial offset *r* ≈ 2 µm via a high‐precision translation stage. The probe laser is then rotated in 15° increments through 360° while both the membrane and the pump laser remain stationary in the laboratory frame. At each azimuth, the Sagnac interferometer detects transient out‐of‐plane surface velocity. Subsequent Fourier analysis of these traces yields the first‐order ZGV resonance frequency as a function of angle, producing a polar pattern that directly identifies the principal crystallographic axes with high contrast.

Dispersion mapping is conducted along each identified principal direction by holding the pump laser fixed and translating the probe laser laterally in 0.2 µm steps over a 20 µm aperture. At each spatial position, the optical delay line is scanned in ∼50 fs increments over a ∼5‐nanosecond window to record the transient surface response. The collected *x–t* matrix was processed with a 2D Fourier transform to generate *f–k* dispersion maps, revealing multiple Lamb branches. These multimodal dispersion data were then input to the SAFE‐GA inversion framework to extract the 3D elastic tensor and membrane thickness with percent‐level precision. To ensure high‐quality input for the SAFE‐GA inversion, the obtained wavenumber–frequency (*k–f*) energy maps undergo systematic signal processing. After transforming the measured spatiotemporal wavefield into the *k–f* domain, filtering is performed in both the wavenumber and frequency domains, followed by normalization and hard thresholding. These steps effectively suppress background noise while preserving only the relevant Lamb‐wave modal energy, yielding clean *k–f* maps that contain essentially pure dispersion information for subsequent inversion. (For further discussion of the optoacoustic experiment, see Figure S9 in Section S4.2).

## Author Contributions

**Pak San Yip**: methodology, validation, investigation, formal analysis. **Yang Lu**: funding acquisition, project administration, resources, writing – review and editing. **Zijian Wang**: methodology, software, investigation, data curation. **Yehai Li**: conceptualization, data curation, supervision, formal analysis, project administration, visualization, funding acquisition, resources, writing – review and editing, methodology. **Jie Huang**: software, data curation, investigation, validation. **Shuchang Zhang**: conceptualization, methodology, software, data curation, investigation, validation, formal analysis, visualization, writing – original draft, writing – review and editing. **Haoyu Cui**: methodology, software, formal analysis, investigation. **Wanglinhan Zhang**: software, methodology, conceptualization, writing – review and editing. **Lin Ye**: funding acquisition, resources, writing – review and editing, project administration. **Guojie Luo**: methodology, data curation, investigation, validation. **Yi He**: conceptualization, methodology, software, investigation, formal analysis, funding acquisition, writing – review and editing, validation, data curation. **Zhongqing Su**: conceptualization, data curation, formal analysis, supervision, funding acquisition, visualization, project administration, resources, writing – review and editing, methodology.

## Conflicts of Interest

The authors declare no conflicts of interest.

## Supporting information




**Supporting File**: advs76472‐sup‐0001‐SuppMat.docx.

## Data Availability

The data that support the findings of this study are available from the corresponding author upon reasonable request.
