# Supplementary material for: Ultrafast Optoacoustics Reveals Intricate 3D Anisotropic Elasticity in Nanocrystalline Membranes
Source: Adv Sci (Weinh). 2026 Jul 7;13(54):e76472. doi: 10.1002/advs.76472 (PMC13339103; doi:10.1002/advs.76472)
Supplement: Supplementary file 1 [file ADVS-13-e76472-s001.docx]

**Supplementary Information**

**For**

***Ultrafast Optoacoustics Reveals Intricate 3D Anisotropic Elasticity in Nanocrystalline Membranes***

Shuchang Zhang^a, #^, Yi He^a, #^, Guojie Luo^a^, Jie Huang^a^, Pak San Yip^b^, Haoyu Cui^a^,

Wanglinhan Zhang^a^, Zijian Wang^c^, Lin Ye^d^, Yang Lu^b, e^, Zhongqing Su^a*^, and Yehai Li^c*^

^a^ Department of Mechanical Engineering

The Hong Kong Polytechnic University, Kowloon, Hong Kong SAR, China

^b^ Department of Mechanical Engineering,

The University of Hong Kong, Hong Kong, Hong Kong SAR, China

^c^ School of Aeronautics and Astronautics

Sun Yat-sen University, Shenzhen, Guangdong, China

^d^ School of Automation and Intelligent Manufacturing,

Southern University of Science and Technology, Shenzhen, Guangdong, China

^e^ Materials Innovation Institute for Life Sciences and Energy (MILES),

HKU-SIRI, Shenzhen, Guangdong, China

^#^ These authors contributed equally: Shuchang Zhang, Yi He.

* To whom correspondence should be addressed.

Email: [Zhongqing.Su@polyu.edu.hk](mailto:Zhongqing.Su@polyu.edu.hk) (Prof. Zhongqing Su, *Ph.D*), [liyh723@mail.sysu.edu.cn](mailto:liyh723@mail.sysu.edu.cn) (Prof. Yehai Li, *Ph.D*)

**This file incorporates:**

**Section 1:** ***SAFE-GA Algorithm for Quantitative Extraction of Elastic Stiffness Constants and Membrane Thickness***

**Section 2: *Photo-Thermal-Elastic Finite Element Simulation Modeling***

**Section 3:** ***Accuracy validation of SAFE-GA Algorithm via Finite Element Simulation***

**Section 4: *Experimental Setup for Sample Fabrication and Ultrafast Optoacoustic Measurement***

**Section 5: *Statistical Analysis of Inverted Experimental Parameters and Sensitivity Analysis***

**References for citations in Supplementary Information**

**1.** **SAFE-GA Algorithm for Quantitative Extraction of Elastic Stiffness Constants and Membrane Thickness**

This Supplementary Information (SI) provides a detailed explanation of the optimized genetic algorithm (GA) framework described in the main text for inverting experimental or simulated $k-f$ dispersion data to extract elastic stiffness constants (*C*) and membrane thickness (*h*). The GA is physics-constrained, multi-objective, and incorporates shear-horizontal (SH) mode suppression to align with out-of-plane (*z*-direction) interferometric detection. It uses a 3D semi-analytical finite-element (SAFE) forward model for dispersion computation. Below, we outline the theoretical foundations, mathematical derivations, algorithmic components, and implementation details, including step-by-step derivations of key formulas. The framework is implemented in MATLAB^®^, with pseudocode referenced for clarity.

**1.1** **Overview of the Genetic Algorithm Framework**

The GA stochastically optimizes a population of candidate parameter sets (chromosomes) to minimize the mismatch between modeled and experimental $k-f$ maps. Each chromosome encodes the parameters: $C_{11}$*,* $C_{12}$*,* $C_{13}$*,* $C_{33}$*,* $C_{44}$ (in GPa), and *h* (in nm). The algorithm enforces physical constraints (e.g., elastic stability) and uses multi-objective fitness balancing structural similarity (SSIM), correlation (Pearson), and amplitude agreement (NRMSE), with penalties for instability.

**Hierarchical Structure:**

- Level 1 (Symmetry Fixing): Prior steps (e.g., ZGV analysis) fix symmetry and principal axes.
- Level 2 (Parameter Inversion): GA optimizes elastic constants and thickness within stability bounds.
- Multiple independent runs ensure robustness, with averaging and outlier rejection.

The GA evolves through generations using selection, crossover, mutation, and elitism, with adaptive strategies to prevent stagnation. SH modes are suppressed in the forward model to mimic *z*-only detection, reducing sensitivity to in-plane polarized constants.

**1.2** **Initialization and Parameter Bounds**

**Parameter Encoding and Bounds Derivation:**
Each chromosome is a vector [$C_{11}$*,* $C_{12}$*,* $C_{13}$*,* $C_{33}$*,* $C_{44}$*, h*]. Bounds are derived from material priors (e.g., for copper: $C_{11}\approx170GPa,h\approx0.5-0.6\mu\text{ }m$ ) and elastic stability conditions for transversely isotropic media:

- Positive definiteness of the stiffness tensor requires:

$$\begin{matrix} C_{11}>\left| C_{12} \right|, C_{44}>0, C_{66}=\frac{C_{11}-C_{12}}{2}>0, \\ \left( C_{11}+C_{12} \right)C_{33}>2C_{13}^{2}. \end{matrix}$$

- Bounds are tightened empirically: e.g., $C_{11}\in[160e9,210e9]Pa,h\in[0.5,0.7]\mu m$. Initialization Derivation:

**Initialization Derivation:**
Population is generated via uniform sampling within bounds, validated by the stiffness matrix eigenvalues:

$$\mathbf{C}=\left[ \begin{matrix} C_{11} & C_{12} & C_{13} & 0 & 0 & 0 \\ C_{12} & C_{11} & C_{13} & 0 & 0 & 0 \\ C_{13} & C_{13} & C_{33} & 0 & 0 & 0 \\ 0 & 0 & 0 & C_{44} & 0 & 0 \\ 0 & 0 & 0 & 0 & C_{44} & 0 \\ 0 & 0 & 0 & 0 & 0 & C_{66} \end{matrix} \right]$$

where eigenvalues *λ* > 0 ensure stability.

**1.3** **Fitness Evaluation with 3D FEM Forward Model and SH** **Suppression**

Fitness quantifies how well a candidate's modeled *k-f* map matches the experimental map. The forward model uses a SAFE approach with through-thickness discretization.

**Forward Model Derivation (SAFE with SH Suppression):**
The elastodynamic equation in variational form is:

$$\int\delta\boldsymbol{\epsilon}^{T}\mathbf{C}\boldsymbol{\epsilon}dV-\omega^{2}\int\rho\delta\mathbf{u}^{T}\mathbf{u}dV=0$$

with assumed form $\mathbf{u}(x,z)=\mathbf{U}(z)e^{i(kz-\omega t)}$. where *ε* is the strain vector, ***C*** is the material stiffness matrix, *ω* is the angular frequency, *ρ* is the density, and *u* is the displacement vector field; *U(z)* is the displacement amplitude vector (or mode shape) as a function of *z,* e is the base of the natural logarithm, *i* is the imaginary unit, *k* is the wavenumber, and *t* is time.
Discretize $z$ into $N\_z$ elements (linear shape functions). The strain-displacement matrix ***B*** for 3D (suppressing $y$-dependence) is derived as:

$$\boldsymbol{\epsilon}=\mathbf{BU}, \mathbf{B}=\left[ \begin{matrix} ikN & 0 & 0 & ikN^{'} & 0 & 0 \\ 0 & 0 & 0 & 0 & 0 & 0 \\ 0 & 0 & dN/dz & 0 & 0 & dN^{'}/dz \\ 0 & dN/dz & 0 & 0 & dN^{'}/dz & 0 \\ dN/dz & 0 & ikN & dN^{'}/dz & 0 & ikN^{'} \\ 0 & ikN & 0 & 0 & ikN^{'} & 0 \end{matrix} \right]$$

where $N,N$ ' are shape functions for nodes 1 and 2.

**Figure S1** illustrates the SAFE workflow used for calculating Lamb-wave dispersion characteristics. It outlines the sequential process, starting from the initialization of anisotropic material stiffness properties, progressing through the application of Floquet–Bloch periodic boundary conditions, and culminating in the formulation and solution of the eigenvalue problem to obtain the wave dispersion data.

**Figure S1** SAFE workflow for Lamb-wave dispersion: from anisotropic stiffness initialization to Floquet–Bloch boundary conditions and eigenvalue solution

Solve for $\omega(k)$ using eigs, then render energy_mask via Gaussian kernels on ( $k,f$ ) grid:

$$energy(f,k)=\sum exp\left( -\frac{\left( f-f_{m} \right)^{2}}{2\sigma_{f}^{2}}-\frac{\left( k-k_{m} \right)^{2}}{2\sigma_{k}^{2}} \right)$$

where energy(*f,k*) is the computed energy density over the grid, *f* is the frequency coordinate on the grid, *k* is the wavenumber coordinate, *fₘ* and *kₘ* are the specific frequency and wavenumber values of the *m*-th mode, *σ_f_* and *σ_k_* are the adaptive standard deviations (bandwidths) of the Gaussian kernels in the frequency and wavenumber directions respectively.

**Figure S2** presents the SAFE forward-modeled dispersion map. Subfigure **(a)** shows the calculated dispersion branches from the 3D model, with each curve representing an eigen-solution for a given wavenumber. Subfigure **(b)** displays the resulting k-f intensity map, where bright ridges trace the modal energy on a normalized color scale.

|   **(a)** |   **(b)** |
| --- | --- |

**Figure S2** SAFE forward-model dispersion map. **(a)**: Calculated dispersion branches from the 3D SAFE model (with through-thickness discretization and SH suppression). Each colored curve is an eigen‑solution for a given longitudinal wavenumber *k* (rad/*µ*m); **(b)**: *k-f* intensity map obtained from the Gaussian kernels of the dispersion branches. Bright, thin ridges trace the observed modal energy, plotted on a normalized amplitude color scale (0–1).

**1.4 SAFE-GA** **pseudocode**

```

% Material Parameter Inversion GA
% Core Algorithm Flow

1. INITIALIZE:
   - Load experimental data (ref_energy_mask)
   - Set target density (rho = 8960)
   - Configure multi-run parameters (50 runs)
```

- Define parameter bounds for *C*_11_, *C*_12_, *C*_13_, *C*_33_, *C*_44_, h

```
2. FOR each run (1 to 50):
   a. INITIALIZE POPULATION:
      - Create 30 random individuals within bounds
      - Ensure physical validity (stability conditions)

   b. FOR each generation (1 to 30):
      i. EVALUATE FITNESS (parallel):
         - Run 3D FEM model with SH suppression
         - Generate energy map
         - Calculate fitness = w1*SSIM + w2*Pearson + w3*(1-NRMSE)
         - Apply physics regularization

      ii. UPDATE BEST SOLUTION
      iii. CHECK EARLY STOPPING (if no improvement in 5 generations)

      iv. EVOLVE POPULATION:
          - Select parents using rank-based selection
          - Create children with blend crossover (80% rate)
          - Mutate with adaptive mutation (25% rate)
          - Apply micro-mutation to top 3 solutions
          - Preserve 2 elites with diversity check

   c. STORE BEST SOLUTION AND METRICS

3. PROCESS RESULTS:
   - Remove outliers using IQR method
   - Calculate average parameters and metrics
   - Generate final model using averaged parameters

4. VISUALIZE:
   - Show experimental vs model energy maps
   - Display parameter distributions
   - Plot residuals

KEY FEATURES:
- Physics-based constraints
- Multi-objective fitness (SSIM + Pearson + NRMSE)
- Adaptive mutation and crossover
- Parallel fitness evaluation
- Multi-run averaging
```

**Figure S3** GA–SAFE inversion workflow: from population initialization to *k-f* fitness evaluation and convergence to optimal elastic tensor and thickness

**Figure S3** outlines the GA–SAFE inversion workflow. It illustrates the sequential process from population initialization, through k-f fitness evaluation, to the convergence that yields the optimal elastic tensor and thickness parameters, which is described in the pseudocode.

**2.** **Photo-Thermal-Elastic Finite Element Simulation Modeling**

This SI provides a detailed expansion of the photo-thermal-elastic finite element simulations described in the main text, conducted using COMSOL Multiphysics^®^ 6.1 to model the ultrafast optoacoustic response of copper membranes. The simulations capture the coupled interactions between optical absorption, thermal diffusion, and elastic wave propagation. Below, we outline the implementation details and workflows for identifying principal elastic axes, performing B-scan simulations, and conducting statistical inversion analysis. All simulations are based on a fully coupled Multiphysics model, with step-by-step derivations of key equations for clarity.

**2.1 Theoretical Foundations and Coupled Multiphysics Model**

The model integrates three physical fields: radiative beam absorption, heat transfer in solids, and solid mechanics (elasticity). These fields are coupled to simulate the generation of coherent acoustic phonons via ultrafast laser pulses.

**2.1.1** **Radiative Beam Absorption**

The laser pulse is modeled as a transient Gaussian energy source absorbed according to the Beer-Lambert law, which describes exponential decay of intensity with depth.

**Beer-Lambert Law:**

The laser pulse is modeled as a transient Gaussian energy source absorbed according to the Beer-Lambert law, which describes exponential decay of intensity with depth.
The incident laser intensity $I_{0}(t)$ at the surface follows a Gaussian temporal profile:

$$I_{0}(t)=I_{\max}exp\left( -\frac{\left( t-t_{0} \right)^{2}}{2\tau^{2}} \right)$$

where $I_{\max}$ is the peak intensity, $t_{0}$ is the pulse center, and $\tau$ is the pulse duration (femtosecond scale).

Absorption in the medium generates a heat source $Q(z,t)$ :

$$Q(z,t)=(1-R)\alpha I_{0}(t)exp(-\alpha z)$$

where $R$ is the surface reflectivity, $\alpha$ is the absorption coefficient, and $z$ is depth.

**2.1.2** **Heat Transfer in Solids**

The absorbed energy induces transient temperature fields, governed by the heat conduction equation with a source term.

**Derivation of Heat Conduction Equation:**

The absorbed energy induces transient temperature fields, governed by the heat conduction equation with a source term.
The temperature $T(\mathbf{r},t)$ satisfies:

$$\rho C_{p}\frac{\partial T}{\partial t}-\nabla\cdot(k\nabla T)=Q(\mathbf{r},t),$$

where $\rho$ is density, $C_{p}$ is specific heat capacity, $k$ is thermal conductivity, and $Q$ is the heat source from absorption.

**2.1.3** **Solid Mechanics (Elasticity)**

Thermoelastic stresses generate elastic waves, solved via the elastodynamic equations coupled to temperature.

**Thermoelastic Elastodynamic Equations:**

The displacement $\mathbf{u}(\mathbf{r},t)$ satisfies:

$$\rho\frac{\partial^{2}\mathbf{u}}{\partial t^{2}}=\nabla\cdot\boldsymbol{\sigma}+\mathbf{f},$$

where $\boldsymbol{\sigma}=\mathbf{C}:\boldsymbol{\epsilon}-\boldsymbol{\beta}\left( T-T_{0} \right)$, with $\mathbf{C}$ the stiffness tensor, $\boldsymbol{\epsilon}=\frac{1}{2}\left( \nabla\mathbf{u}+(\nabla\mathbf{u})^{T} \right)$ the strain, $\boldsymbol{\beta}$ the thermal stress tensor (e.g., $\beta_{ij}=C_{ijkl}\alpha_{kl},\alpha$ thermal expansion), and **f** body forces (negligible).

Coupling arises from the temperature-dependent stress term, derived from the free energy expansion:

$$\boldsymbol{\sigma}=\frac{\partial F}{\partial\boldsymbol{\epsilon}}=\mathbf{C}:\boldsymbol{\epsilon}-\boldsymbol{\beta}\Delta T.$$

Boundary conditions: Traction-free surfaces ( $\boldsymbol{\sigma}\cdot\mathbf{n}=0$ ) at membrane interfaces; periodic or perfectly matched layers (PML) laterally to absorb outgoing waves and prevent reflections.

**2.1.4 Mesh and Time Integration**

The computational domain is defined as either a 3D membrane sector for single‑crystal simulations or a 2D membrane section for transversely isotropic cases, consistent with the optoacoustic modeling framework. For single‑crystal ZGV in 3D, a 90°/60° sector with symmetry boundary conditions is used and meshed by a circular‑arc swept strategy; the through‑thickness element size is 35 nm, and the 90° arc is discretized at 2.5° per segment to resolve the in‑plane angular dependence of the modes. For the transversely isotropic 2D model, a structured quadrilateral mesh with a uniform in‑plane element size of 30 nm is employed across the domain to ensure consistent resolution of guided‑wave features. Linear systems arising at each time step are solved using the MUMPS (MUltifrontal Massively Parallel Sparse) direct solver, time integration adopts a generalized‑α with fixed stepping (Δ*t*=1 ps, tolerance 1e−6) to maintain stability for the coupled photo‑thermal‑elastic problem.

**2.2 Material Anisotropy Models**

Material properties are encoded via symmetry-specific stiffness tensors.

**2.2.1 Single-Crystal Copper (100)**

Three independent constants: *C*_11_, *C*_12_, *C*_44_. Tensor in Voigt notation:

$$\mathbf{C}=\left[ \begin{matrix} C_{11} & C_{12} & C_{12} & 0 & 0 & 0 \\ C_{12} & C_{11} & C_{12} & 0 & 0 & 0 \\ C_{12} & C_{12} & C_{11} & 0 & 0 & 0 \\ 0 & 0 & 0 & C_{44} & 0 & 0 \\ 0 & 0 & 0 & 0 & C_{44} & 0 \\ 0 & 0 & 0 & 0 & 0 & C_{44} \end{matrix} \right]$$

Derived from cubic invariance under ${90}^{\circ}$ rotations.

**2.2.2 Single-Crystal Copper (111)**

Six independent constants: *C*_11_, *C*_12_, *C*_13_, *C*_33_, *C*_44_, *C*_15_. with *C*_66_ $=($ *C*_11_ - *C*_12_ $)/2$. Tensor:

$$\mathbf{C}=\left[ \begin{matrix} C_{11} & C_{12} & C_{13} & 0 & C_{15} & 0 \\ C_{12} & C_{11} & C_{13} & 0 & -C_{15} & 0 \\ C_{13} & C_{13} & C_{33} & 0 & 0 & 0 \\ 0 & 0 & 0 & C_{44} & 0 & {-C}_{15} \\ C_{15} & {-C}_{15} & 0 & 0 & C_{44} & 0 \\ 0 & 0 & 0 & {-C}_{15} & 0 & C_{66} \end{matrix} \right]$$

Derived from cubic invariance under ${60}^{\circ}$ rotations.
**2.2.2 Transversely Isotropic Copper**

Five constants: *C*_11_, *C*_12_, *C*_13_, *C*_33_, *C*_44_, with *C*_66_ $=($ *C*_11_ - *C*_12_ $)/2$. Tensor:

$$\mathbf{C}=\left[ \begin{matrix} C_{11} & C_{12} & C_{13} & 0 & 0 & 0 \\ C_{12} & C_{11} & C_{13} & 0 & 0 & 0 \\ C_{13} & C_{13} & C_{33} & 0 & 0 & 0 \\ 0 & 0 & 0 & C_{44} & 0 & 0 \\ 0 & 0 & 0 & 0 & C_{44} & 0 \\ 0 & 0 & 0 & 0 & 0 & C_{66} \end{matrix} \right]$$

This model in-plane isotropy with out-of-plane anisotropy.

**2.3 Identification of Principal Elastic Axes Using ZGV Resonances**

To determine the principal axes of an anisotropic membrane, excite broadband guided modes using a Gaussian laser pulse as the broadband source and record the co‑located out‑of‑plane velocity v_z(t); compute the magnitude spectrum S(f) from the FFT of v_z(t) and identify the ZGV resonance as the dominant spectral peak associated with zero group velocity (per the prior derivation). Repeat this simulation while rotating the in‑plane angle θ (by rotating the sampling point), then fit the angle‑dependent ZGV frequency with a cubic‑symmetry form f_ZGV(θ) ≈ f_0 + Δf cos(4θ). The extrema of this fit mark the in‑plane principal axes (e.g., near the ⟨100⟩ directions for cubic textures), and the modulation amplitude Δf provides a quantitative measure of the elastic anisotropy.

**2.4 Spatiotemporal Scan Simulation Workflow**

**2.4.1 Spatiotemporal Scan Simulation Setup**

A one‑dimensional B‑scan is simulated along a line aligned with the principal axis, sampling x from 0 *µ*m to +20 *µ*m at Δx = 0.2 *µ*m and recording the out‑of‑plane wavefield at temporal rate fs over a window long enough to capture the guided‑wave coda. The space–time data are loaded from the measurement table, converted to double precision, and mirrored laterally to extend the effective aperture and suppress edge artifacts before analysis, ensuring the spatial grid and observation window support accurate frequency–wavenumber mapping. Each spatial trace is band‑pass filtered with a low‑order Butterworth filter to isolate the ZGV‑relevant band and improve signal‑to‑noise while maintaining a flat passband and coherent phase across the scan line for subsequent spectral estimation. The raw and filtered B‑scans are visualized as images with axes calibrated in micrometers and nanoseconds to verify data quality, modal content, and the absence of early boundary reflections within the observation window prior to spectral processing. A 2D Fourier transform is then applied to obtain the dispersion map, which reveals the Lamb‑mode ridges and enables direct identification of mode families and ZGV features in frequency–wavenumber space. Precomputed symmetric and antisymmetric dispersion curves from a forward model are overlaid on for validation, enabling quantitative comparison between measured ridges and theoretical branches across the scanned k–f region.

**2.4.2 Material parameters**

| **Parameter** | **Symbol** | **Value** | **Units** |
| --- | --- | --- | --- |
| Density | *ρ* | 8960 | kg/m³ |
| Absorption Coefficient (Optical) | *α* | ~6.37 × 10⁷ | m⁻¹ |
| Specific Heat Capacity | *Cp* | 385 | J/kg·K |
| Thermal Conductivity | *k* | 400 | W/m·K |

For single-crystal copper (100): *C*_11_=168.3GPa, *C*_12_=122.1GPa, *C*_44_=75.7GPa.

For single-crystal copper (111): *C*_11_=221.07GPa, *C*_12_=104.88GPa, *C*_13_=87.55GPa, *C*_33_=238.39GPa, *C*_44_=40.77GPa, *C*_15_=24.49GPa.

For transversely isotropic copper: *C*_11_=160GPa, *C*_12_=70GPa, *C*_13_=80GPa, *C*_33_=180GPa, *C*_44_=75GPa.

**2.4.3 Compute environment (simulation server/workstation)**

- CPU: AMD^®^ Ryzen 9 9950X, 16 cores (Granite Ridge, 4 nm), SMT on; strong single/multi‑thread for time/frequency domain solvers.
- Memory: 192 GB DDR5 dual‑channel (DDR5‑3588 effective).
- Motherboard: MSI^®^ MAG B650M MORTAR WIFI (B650), PCIe 5.0.
- GPU: NVIDIA^®^ GeForce RTX 4060, 8 GB GDDR6; used for visualization and optional FFTs; CPU runs primary solvers.
- Storage: PCIe Gen4 NVMe for checkpoints and k–f datasets.

Compute runtime summary (this system):

- 3D transient (5 ns physical time, 1 ps step): ~25 hours.
- 2D transient (5 ns, 1 ps step): ~2 hours.
- Genetic algorithm inversion: ~15 minutes per run (per seed/config), parallelized across cores when sweeping.
  2.5 **Supplementary Simulation Results**

**Figure S4** systematically illustrates the spatiotemporal evolution of the multi-physical photo-thermal elastic fields in a membrane following laser irradiation. The figure presents the sequence in three main temporal stages: Excitation Stage: At the very early time of 1 picosecond (ps), subfigure **(a)** shows the spatial distribution of the absorbed optical intensity (units: W/m²), which serves as the initial heat source for all subsequent processes. Thermal Diffusion and Expansion Stage: By 5 ps, subfigure **(b)** depicts the resulting transient temperature field (units: K). The non-uniform temperature distribution induces localized thermal expansion, generating the driving force for elastic waves. Elastic Wave Propagation Stage (Panels c-f): The subsequent series of subfigures **(c-f)** tracks the propagation of the induced elastic waves, characterized by the particle velocity distribution (units: m/s). The snapshots at 500 ps, 1000 ps, 2000 ps, and 3000 ps clearly show the dynamic evolution of the wavefronts, including their expansion, reflection, and interference within the membrane. Collectively, the figure provides a coherent visualization of the complete physical process, from optical energy absorption to thermal conversion, and finally to the generation and propagation of ultrasonic elastic waves.

**(a)**

**(b)**

**(c)**

**(d)**

**(e)**

**(f)**

**Figure S4** Spatiotemporal photo‑thermal‑elastic fields in the membrane. (a) absorbed optical intensity at 1 ps, unit W/m^2. (b) temperature at 5 ps, unit K. (c–f) particle velocity distributions at 500, 1000, 2000, and 3000 ps, respectively, unit m/s.

**Figure S5** compares simulated acoustic responses in two copper membranes: **transversely isotropic** **(Fig. S5 (a) and (b))** and **single-crystal (Fig. S5 (c) and (d))**. The simulated time-domain signals at two positions show that both the single-crystal and transversely isotropic cases exhibit high signal-to-noise ratios. The single-crystal case displays faster wave propagation (higher wave speeds) than the transversely isotropic case.

|   **(a)** |   **(b)** |
| --- | --- |
|   **(c)** |   **(d)** |

**Figure S5** Simulated time‑domain and B‑scan responses in copper membranes with different anisotropy. **(a)** time domain signal of transversely isotropic Cu; **(b)** B‑scan (x–t) map for transversely isotropic Cu; **(c)** time domain signal of monocrystal Cu; **(d)** B‑scan (x–t) map for monocrystal Cu;

**3. Accuracy validation of SAFE-GA Algorithm via Finite Element Simulation**

**3.1 Finite Element Simulation** **Inversion Results**

To validate the accuracy of the method, the SAFE‑GA is first validated in simulations before being applied to experiments. The elastic stiffness matrix for non-principal directions becomes prohibitively complex—featuring strong anisotropic coupling and skew energy-flux components. To simplify the inversion and improve stability, spatiotemporal imaging of Lamb waves in copper membranes is performed along the identified principal directions. This condition ensures that the inversion problem remains computationally tractable. The wave dispersion relationship extracted from these *k–f* spectra are analyzed using the SAFE–GA: the plate model enforces traction–free boundaries and geometry, while the GA explores a multimodal objective landscape caused by branch crossings, partial band coverage, and mode aliasing, which ensures stable recovery even when only a subset of Lamb modes can be extracted from the *k–f* spectra.

For the monocrystalline Cu (100) membrane in simulation, inversion targets the cubic elastic constants *C*_11_, *C*_12_, *C*_44_ and the thickness. To mitigate the intrinsic variability of the genetic algorithm, the inversion parameters are estimated from 50 independent GA realizations. Averaging across multiple runs smooths the out stochastic fluctuations arising from random initial populations, crossover, and mutation, ensuring that the final estimates are both robust and reproducible despite the stochastic nature of GA search. The distributions are narrow, nearly symmetric, and centered on ground truth, with relative errors of approximately 4.3% (*C*_11_), 2.2% (*C*_12_), 3.0% (*C*_44_), and 1.4% (thickness) (**Fig. S6 (a)**). Although the cubic tensor introduces stronger parameter coupling than isotropic plates, jointly fitting multiple branches over a broad spectral window yields precise recovery without reliance on any single feature. For the transversely isotropic copper membrane, the inversion estimated *C*_11_, *C*_12_, *C*_13_, *C*_33_, *C*_44_ and thickness with similarly tight posteriors, reflecting reduced parameters coupling from in–plane isotropy and complementary flexural versus extensional sensitivities (**Fig. S6 (b)**). In the low frequency–thickness limit, the A_0_ mode of Lamb waves (the fundamental antisymmetric mode) chiefly constrains shear stiffness *C*_44_, whereas the S_0_ mode of Lamb waves (the fundamental symmetric mode) constrains in–plane longitudinal stiffness *C*_11_; at larger frequency–thickness, higher–mode spacing and cut–off behavior provide the most reliable thickness and *C*_33_ information. Dispersion curves computed from ensemble–mean parameters closely follow the simulated *k*–*f* intensity (**Figs. S6 (c) (d)**)—reproducing slopes, curvature, and cut–offs—and the residuals are commensurate with posterior spreads, indicating appropriate regularization and an unbiased, stable inversion.

|   **(a)**    **(b)** | |
| --- | --- |
|   **(c)** |   **(d)** |

**Figure S6** GA‑enabled inversion simulation results: (a) estimation distributions for elastic constants and thickness of monocrystal copper; (b) estimation distributions for elastic constants and thickness of transversely isotropic copper; (c) frequency–wavenumber dispersion with fitted Lamb branches of monocrystal copper; (d) frequency–wavenumber dispersion with fitted Lamb branches of transversely isotropic copper.

**3.2 Finite Element Simulation Inversion Results Statistical analysis**

Based on the statistical results, the inversion algorithm demonstrates robust performance in recovering the single-crystal elastic constants and thickness, with all relative errors maintained below 5%, indicating a high consistency between the inverted parameter set and the predefined true values (**Table S1**). Specifically, the thickness exhibits the highest recovery accuracy with a relative error of only 1.42%. The elastic constants *C*_12_ and *C*_44_ are also accurately determined, with relative errors of 2.18% and 3.04%, respectively. The error for *C*_11_ is slightly higher at 4.28% but remains within an acceptable range. The distributions of the estimated values, represented by the 95% confidence intervals, are reasonable and show no abnormal dispersion.

Analysis of the parameter correlations reveals a distinct coupling pattern (**Table S2**). A very strong positive correlation (coefficient of 0.861) exists between the elastic constant *C*_11_ and the Thickness. This indicates that within the inversion model, an increase in longitudinal stiffness (*C*_11_) and an increase in sample thickness produce highly similar physical signal responses, making it mathematically challenging to resolve these two parameters independently and uniquely. Furthermore, moderate positive correlations are observed between *C*_12_ and *C*_11_ (0.689) and between *C*_12_ and thickness (0.682). *C*_44_ also shows a moderate correlation primarily with Thickness (0.608). Together, these correlations define the primary structure of the parameter uncertainty space, with the strong coupling between *C*_11_ and Thickness being the most critical factor affecting the uniqueness of the parameter solution.

**Table S1** Statistical summary of inverted parameters of monocrystalline Cu (100) membrane

| Parameter | Mean±Std (GPa/nm) | True Value | Absolute Error | Relative Error (%) |
| --- | --- | --- | --- | --- |
| *C*_11_ | 161.1 ± 3.205 | 168.3 | 7.2033 | 4.28% |
| *C*_12_ | 124.76 ± 3.594 | 122.1 | 2.661 | 2.17% |
| *C*_44_ | 78.003 ± 1.890 | 75.7 | 2.3027 | 3.04% |
| Thickness | 542.21 ± 4.888 nm | 550 | 7.7931 | 1.42% |

**Table S2.** Correlation matrix of inverted parameters of monocrystalline Cu (100) membrane

|  | *C*_11_ | *C*_12_ | *C*_44_ | Thickness |
| --- | --- | --- | --- | --- |
| *C*_11_ | 1 | 0.68906 | 0.13473 | 0.86134 |
| *C*_12_ | - | 1 | 0.18654 | 0.68206 |
| *C*_44_ | - | - | 1 | 0.60774 |
| Thickness | - | - | - | 1 |

The inversion of transversely isotropic copper membrane demonstrates remarkable accuracy (**Table S3**), with relative errors below 4% for all parameters. Particularly noteworthy is the exceptional precision achieved for *C*_13_ (0.65% error) and *C*_44_ (0.65% error), indicating strong sensitivity of the dispersion characteristics to these parameters. The thickness estimation shows sub-1% error (0.94%), validating the method's capability for precise dimensional characterization. The correlation matrix reveals significant inter-parameter relationships (**Table S4**). The strongest correlation observed is between *C*_33_ and thickness (r = 0.927, p < 0.001*), indicating these parameters exhibit complementary effects on the dispersion curves. This high correlation suggests that variations in thickness can be partially compensated by adjustments in *C*_33_, presenting a potential identifiability challenge that the multi-objective fitness function successfully resolves through the incorporation of multiple dispersion branches. Moderate positive correlation exists between *C*_11_ and *C*_44_ (r = 0.503, p < 0.001*), consistent with their shared dependence on in-plane wave propagation characteristics.

**Table S3** Statistical summary of inverted parameters from 50 GA runs of transversely isotropic copper membrane

| Parameter | Mean±Std (GPa/nm) | True Value | Absolute Error | Relative Error (%) |
| --- | --- | --- | --- | --- |
| *C*_11_ | 163.31 ± 5.09 | 160.00 | 3.31 | 2.07% |
| *C*_12_ | 72.40 ± 3.89 | 70.00 | 2.40 | 3.43% |
| *C*_13_ | 80.52 ± 4.06 | 80.00 | 0.52 | 0.65% |
| *C*_33_ | 173.01 ± 5.04 | 180.00 | 6.99 | 3.88% |
| *C*_44_ | 75.49 ± 4.63 | 75.00 | 0.49 | 0.65% |
| Thickness | 544.82 ± 6.28 nm | 550.00 nm | 5.18 nm | 0.94% |

**Table S4** Correlation matrix of inverted parameters from 50 GA runs of transversely isotropic copper membrane

|  | *C*_11_ | *C*_12_ | *C*_13_ | *C*_33_ | *C*_44_ | Thickness |
| --- | --- | --- | --- | --- | --- | --- |
| *C*_11_ | 1 | 0.14657 | -0.25922 | -0.35581 | 0.50297 | -0.13898 |
| *C*_12_ | - | 1 | -0.003696 | -0.0060997 | -0.095927 | -0.036296 |
| *C*_13_ | - | - | 1 | 0.16775 | -0.29075 | 0.12371 |
| *C*_33_ | - | - | - | 1 | -0.28941 | 0.9274 |
| *C*_44_ | - | - | - | - | 1 | 0.067708 |
| Thickness | - | - | - | - | - | 1 |

**4. Experimental Setup for Ultrafast Optoacoustic Measurement**

This SI provides a detailed description of the experimental procedures for fabricating freestanding copper membranes and performing ultrafast optoacoustic measurements, as outlined in the main text. It includes step-by-step protocols, theoretical foundations, equipment specifications, and optimization strategies to ensure reproducibility and high-quality data acquisition. The methods are designed to enable precise characterization of membrane thickness, elastic properties, and anisotropy through guided acoustic wave analysis.

**4.1 Sample Fabrication Process**

Freestanding copper membranes are fabricated using a combination of electron-beam sputtering deposition and selective etching techniques. The process is optimized for producing ultrathin (nanometer-scale) membranes with controlled microstructure and high uniformity. Below, we detail each step, including rationale and parameters.

**4.1.1 Substrate Preparation**

Commercial SiN membranes (Norcada^®^ NX5200D) and single‑crystal Si(100) membranes (Norcada^®^ SUF521.6D) are used as substrates.

**4.1.2 Electron-Beam (E-Beam) Sputtering Deposition**

Copper films are deposited in an E‑beam sputtering system (Denton Explorer^®^ E-beam Deposition System) under high vacuum (< 5 × 10⁻⁷ Torr) using a high‑purity Cu target. A thin chromium adhesion layer (~5 nm) is deposited first, followed by the copper layer (~550 nm). Thickness is monitored in situ by quartz crystal microbalance and verified ex situ.

**4.1.3 Selective Etching and Membrane Release**

Membranes are released by ICP‑RIE (Phantom RIE ICP, TRION TECHNOLOGY^®^) under optimized CF₄/O₂ conditions, followed by wet etching (Cr etch 210, MicroChemicals^®^ GmbH) to remove the chromium adhesion layer without damaging copper.

**4.1.4 Ex-Situ Copper Film Thickness Measurement on Glass Witness**

For each deposition run, a glass witness slide is positioned alongside the membrane samples and coated simultaneously to offer an ex-situ reference for the copper film thickness. Upon completion of deposition, the thickness of the copper on the witness is quantified by stylus profilometry (Bruker DektakXT^®^), wherein a line scan is made across a distinct step edge defined by masking or lift-off. The measured vertical displacement at the step, as illustrated in **Figure S7**, is taken as the film thickness. These profilometer step-height data provided a direct calibration for the deposition rate and reproducibility, serving to verify or adjust the nominal thickness values reported by the in-situ quartz crystal microbalance. Instrument accuracy is maintained through routine checks of stylus and vertical calibration before measurement. The representative results for Cu^SiN^ and Cu^Si^ membranes, shown in **Figure S7 (a) and (b)**, confirm step heights of 524.5 nm and 609.7 nm, respectively. Based on this result, after subtracting the chromium layer thickness, the thicknesses of the Cu^SiN^ and Cu^Si^ films are approximately 519.5 nm and 604.7 nm, respectively.

|   **(a)** |   **(b)** |
| --- | --- |

**Figure S7** Stylus profilometry step‑height measurements on coated glass witnesses: **(a)** Cu^SiN^ membrane 524.5 nm, **(b)** Cu^Si^ membrane with 609.7 nm

**4.1.5 XRD Crystal Texture Analysis**

X‑ray diffraction (XRD) characterization results corroborate the microstructural and geometric interpretations drawn from ultrafast optoacoustic analysis. The Cu^SiN^ membrane exhibits a nearly isotropic diffraction pattern, aligning with the quasi‑isotropic elastic tensor inferred from ZGV analysis (**Figure S8(a)**). These observations indicate that substrate‑driven deposition conditions strongly govern the degree of elastic anisotropy in ultrathin copper membranes. By contrast, results reveal a pronounced (111) texture in the Cu^Si^ membrane, consistent with the elevated shear rigidity *C*_44_ and anisotropic elastic response obtained from SAFE–GA inversion (**Figure S8(b)**). The strong (111) orientation increases effective out‑of‑plane longitudinal stiffness^1, 2^ (higher *C*_33_) and produces measurable in‑plane anisotropy, which is reflected in both ZGV frequency shifts and guided wave dispersion features.

|   **(a)** |   **(b)** |
| --- | --- |

**Figure S8** XRD of ultrathin membranes: (a) Cu^SiN^ with near isotropic diffraction consistent with quasi-isotropic stiffness; (b) Cu^Si^ with pronounced Cu (111) texture indicating anisotropic elasticity.

**4.2 Ultrafast Optoacoustic Measurement System**

The ultrafast optoacoustic measurement system is configured as a femtosecond pump–probe setup combined with a Sagnac interferometer for high-sensitivity, non-contact probing of guided acoustic waves propagating in nanoscopic structures such as freestanding membranes^3^ (**Fig. S9**).

**4.2.1 Pump Beam Configuration**

The pump beam is generated by a commercial ultrafast laser source (Amplitude®, Tangor 100 IR with Compress 10) producing p-polarized femtosecond pulsed infrared beams at 1,030 nm wavelength, 50 fs pulse duration, and 807 kHz repetition rate, with an average power of 10 W. The beam is split via a nonpolarizing beam splitter (NPBS-1, 9:1 transmission-to-reflection) into a high-power transmitted portion (9 W) used as pump and a lower-power reflected portion (1 W) reserved as a probe. The pump beam is modulated by an acousto-optic modulator (AOM, G&H®, AOMO 3080-1990), driven by an arbitrary waveform generator (RIGOL®, DG1022) supplying a continuous square-wave signal with 50% duty cycle. A beta barium borate (BBO) nonlinear crystal (EKSMA Optics®, BBO-651H) frequency-doubles part of the pump IR photons to 515 nm. The dichroic mirror-1 (DM-1) filters out residual 1,030 nm light, allowing primarily the 515 nm beam to proceed. The pump beam power is attenuated down to approximately 2 mW at the sample surface to ensure thermoelastic generation without inducing damage.

**4.2.2 Probe Beam and Sagnac Interferometer**

The probe beam, originating from the reflected 1 W IR beam, is temporally delayed via an optical delay line (ODL, OptoSigma®, HST(GS)-200-0B) with adjustable free-space optical path length from 0 to 4.7873 ns. After the ODL, the probe enters a custom Sagnac interferometer mounted on a motorized yz‑motion stage (OptoSigma, OSMS‑20‑35) for stable alignment and reproducible overlap on the sample. The interferometer comprises two half‑wave plates (HWP‑1, HWP‑2), two quarter‑wave plates (QWP‑1, QWP‑2), two polarizing beam splitters (PBS‑1, PBS‑2), one nonpolarizing beam splitter (NPBS‑2), a pair of relay/focusing lenses (CL‑1, CL‑2), and a balanced photodetector (NewOpto, OPM‑220In‑B). The Sagnac configuration converts polarization changes into phase‑ and displacement‑sensitive readout with intrinsic common‑path noise rejection, enabling detection of guided acoustic waves on the membrane without quoting numeric sensitivity figures. The probe power is kept low to avoid perturbing thermoelastic generation while maintaining adequate detection sensitivity. The pump and probe beams are combined by a dichroic mirror (DM‑2) and focused onto the sample using a high numerical‑aperture microscope objective (ZEISS, EC Epiplan‑Neofluar 100×/0.9 BD DIC M27). The interferometric output is demodulated by a lock‑in amplifier (SRS, SR830) referenced to the pump modulation to extract weak guided‑wave signals from noise without stating time‑constant or bandwidth numbers. The entire setup is installed on a vibration‑isolated optical table (Newport, RS 4000TM) with pneumatic isolators (Newport, I‑2000 Stabilizer) and enclosed to suppress ambient light and environmental disturbances. The system’s temporal resolution is governed by the probe pulse duration and the delay‑line increment, while the effective acoustic measurement bandwidth is set by the optomechanical detection chain.

**Figure S9** Ultrafast pump–probe and Sagnac interferometer optical bench illuminated at 515 nm for GHz optoacoustic measurements

**4.2.3 Supplementary experimental results**

Initial spatial alignment of pump and probe beams is performed using a CCD camera-assisted optical path visualization system combined with piezo-controlled mirrors, optimized to maximize interferometric signal amplitude. To analyze ZGV resonances and elastic anisotropy, the probe is rotated through 360° with angular steps of 15°. Velocity signals are collected at fixed probe points and analyzed by fast Fourier transform to extract spectral features for each orientation (**Figure S10(a) and (b)**). For B-scan acquisition, the probe beam is scanned linearly over sample regions of 20 μm with step sizes between 0.2μm using a high-precision translation stage. Time-resolved out-of-plane velocity signals are recorded at each spatial location with a high temporal sampling rate (**Figure S11(a) and (b)**). All measurements are conducted in controlled laboratory conditions with environmental temperature maintained between 20–25°C and relative humidity below 50%. The setup is vibration-isolated to reduce noise.

**(a)**

**(b)**

**Figure S10** Angle‑resolved ZGV spectra of copper membranes. **(a)** Membrane deposited on amorphous SiN: spectra for 0°–345° in 15° steps, showing near‑circular angular response with prominent ZGV peaks. **(b)** Membrane deposited on single‑crystal Si(100): corresponding angle sweep under identical conditions, exhibiting similar circularity and distinct absolute ZGV frequencies relative to SiN.

**(a)**

**(b)**

**Figure S11** Time‑domain traces at multiple offsets for copper membranes. **(a)** Cu^SiN^ Membrane deposited on amorphous SiN: *v_z_(t)* recorded at offsets 0–18 μm in 3 μm steps, showing strong early‑time response near the source with rapid decay at larger offsets. **(b)** Cu^Si^ Membrane deposited on single‑crystal Si(100): corresponding *v_z_(t)* series under identical conditions, exhibiting similar arrival ordering and offset‑dependent attenuation.

**Figure** **S12** presents the experimental results of parameter inversion enabled by the SAFE-GA. The SAFE–GA posterior distributions of Cu^SiN^ membrane are tight and centered near *C*_11_ = 175.22 GPa, *C*_12_ = 76.41 GPa, *C*_13_ = 84.75 GPa, *C*_33_ = 177.08 GPa, *C*_44_ = 43.93 GPa, and *h* = 529.77 nm (**Fig. S12(a)**), with *C*_11_ ≈  *C*_33_，indicating quasi–isotropic in–plane response consistent with weak or random texture obtained from XRD results. For the Cu^Si^ membrane, the SAFE–GA posteriors converge on *C*_11_ = 166.74 GPa, *C*_12_ = 79.22 GPa, *C*_13_ = 74.06 GPa, *C*_33_ = 189.92 GPa, *C*_44_ = 133.58 GPa, and *h* = 623.12 nm as labeled in the violin plots (**Fig. S12(b)**), with a substantially higher *C*_33_ compared to the in-plane longitudinal modulus *C*_11_.

**(a)**

**(b)**

**Figure S12** GA‑enabled inversion experimental results: **(a)** estimation distributions for elastic constants and thickness of Cu^SiN^ membrane; **(b)** estimation distributions for elastic constants and thickness of Cu^Si^ membrane

**4.3 TEM Analysis Sample Preparation and Imaging**

To enable high-resolution transmission electron microscopy (TEM) characterization of the Cu^Si^ film microstructure, focused ion beam (FIB) milling was performed on the Cu film deposited on single-crystal Si(100) substrate prior to membrane release. **Figure S13** illustrates the preparation process using the Thermo Scientific Scios 2 HiVac DualBeam FIB/SEM system. The high-magnification plan-view SEM image (**Fig. S13(a)**) shows equiaxed grains with an average grain size of ~100 nm, consistent with the in-plane elastic isotropy and the transversely isotropic elastic constants obtained from the SAFE-GA inversion. The top-view SEM image of the platinum protective strip deposited on the selected area prior to milling is presented in **Fig. S13(b)**. The SEM image of the excavated site with trenches created by FIB milling is shown in **Fig. S13(c)**. The SEM image of the TEM-ready cross-sectional lamella, revealing the uniform Cu layer, sharp Cu/Si interface, and the protective Pt capping layer on top, is displayed in **Fig. S13(d)**.

**Figure S14** presents the TEM results of the as-prepared cross-sectional lamella using the JEOL JEM-F200. Low-magnification images **(a, b)** reveal columnar-like grains with a moderate number of nanotwined grains scattered throughout. In the typical nanotwinned region **(c)**, the SAED pattern from the red-circled area **(d)** shows two distinct sets of diffraction spots, clearly confirming coherent nanotwins inside the (111) matrix. The SAED pattern from a twin-free region **(e, f)** also can be determined to the (111) orientation, which reveals the strong and dominant (111) texture throughout the film, consistent with XRD results. **Figure S14 (g)** is the high magnification of nanotwins showing sharp, straight coherent twin boundaries, and **(h)** gives the overall lamella view confirming uniform microstructure. These TEM images and SAED patterns provide direct evidence that matches our earlier hypotheses. Based on these observations, we hypothesize that the increase in *C*₃₃, together with the remarkably high *C*₄₄ in the Cu^Si^ membrane, may arise from the strong (111) out-of-plane texture combined with the moderate density of coherent nanotwins observed in our TEM characterization. At the same time, other mechanisms—such as possible metastable FCC/HCP phase coexistence or additional defect structures—cannot be ruled out and may also contribute to the elevated stiffness.

|   **(a)** |   **(b)** |
| --- | --- |
|   **(c)** |   **(d)** |

**Figure S13** FIB preparation and multi-scale electron microscopy characterization of the Cu film deposited on single-crystal Si(100) substrate. **(a)** High-magnification plan-view SEM image showing the nanocrystalline surface morphology with average grain size ~100 nm. **(b)** Top-view SEM image of the platinum strip deposited on the surface of selected location to be cut **(c)** SEM image of the excavated site with trenches created by FIB milling. **(d)** SEM image of the TEM ready cross-sectional lamella with thickness of about 150 nm, revealing the uniform Cu layer, sharp Cu/Si interface, and the protective Pt capping layer on top. Scale bars: 500 nm (a), 5 μm (b), 3 μm (c), 2 μm (d).

|   **(a)** | **(b)** |
| --- | --- |
|   **(c)** |   **(d)** |
| .  .  **(e)** |   **(f)** |
|   **(g)** |   **(h)** |

**Figure S14** Transmission electron microscopy (TEM) characterization of the Cu film deposited on Si(100). **(a)** TEM image showing densely packed columnar-like grains with the presence of nanotwins. **(b)** TEM image of another region with densely packed columnar-like grains, also containing a certain number of nanotwins. **(c)** Magnified TEM image of the twins in **(a)**. **(d)** Selected-area electron diffraction (SAED) pattern acquired from the red-circled area in **(c)**, showing two distinct sets of diffraction spots that confirm the presence of coherent nanotwins within the (111) matrix. **(e)** TEM image of another region, with the red circle indicating a twin-free area. **(f)** SAED pattern acquired from the red-circled twin-free region in **(e)**, can be identified to the (111) orientation. **(g)** High-magnification TEM image showing a sharp and straight coherent nanotwin boundary. **(h)** Overall low-magnification view of the FIB-prepared lamella.

**5. Statistical Analysis of Inverted Experimental Parameters and Sensitivity Analysis**

**5.1 Statistical Summary of Inverted Parameters**

To rigorously assess the stability, reproducibility, and robustness of the SAFE-GA inversion, a comprehensive statistical analysis was performed based on 50 independent GA runs for both the Cu^SiN^ and Cu^Si^ membranes after IQR-based outlier removal. The statistical summaries of the inverted parameters (mean ± standard deviation) are presented in **Tables S5** (Cu^SiN^) and **S7** (Cu^Si^). For the Cu^SiN^ membrane, all parameters show narrow distributions with relative standard deviations below 6%. Similar tight distributions are observed for the Cu^Si^ membrane, confirming the high reproducibility of the multi-run inversion procedure. The corresponding pairwise correlation matrices are shown in Tables **S6** (Cu^SiN^) and **S8** (Cu^Si^). In both membranes, the strongest correlation is found between C₃₃ and thickness (r = 0.93841 for Cu^SiN^ and r = 0.96505 for Cu^Si^), which is physically expected from plate-wave dispersion theory. Moderate correlations are also present between *C*₁₁ and *C*₄₄, while correlations involving *C*₁₂ and *C*₁₃ remain relatively weak. These results indicate that although certain parameter pairs exhibit coupling (particularly C₃₃–thickness), the multi-objective fitness function and ensemble averaging effectively constrain the solution space, yielding stable and reliable inversion outcomes with low uncertainty for all elastic constants and thickness. All six parameters were inverted simultaneously by the SAFE-GA algorithm.

The Pearson correlation coefficient $r$ between any two variables $X$ and $Y$ is calculated as:

$$r_{XY}=\frac{\sum_{i=1}^{n} \left( x_{i}-\bar{x} \right)\left( y_{i}-\bar{y} \right)}{\sqrt{\sum_{i=1}^{n} \left( x_{i}-\bar{x} \right)^{2}\sum_{i=1}^{n} \left( y_{i}-\bar{y} \right)^{2}}}$$

where $n=50$, $\bar{x}$ and $\bar{y}$ are the sample means of $X$ and $Y$, respectively. This coefficient quantifies the strength and direction of the linear relationship between each pair of parameters, ranging from -1 (perfect negative correlation) to +1 (perfect positive correlation), with 0 indicating no linear relationship.

**Table S5** Statistical summary of inverted parameters of Cu^SiN^ membrane.

| **Parameter** | **Mean±Std (GPa/nm)** |
| --- | --- |
| ***C*_11_** | **175.22 ± 5.40** |
| ***C*_12_** | **76.41 ± 9.97** |
| ***C*_13_** | **84.75 ± 6.24** |
| ***C*_33_** | **177.08 ± 6.47** |
| ***C*_44_** | **43.93 ± 1.67** |
| **Thickness** | **529.77 ± 9.21 nm** |

**Table S6** Correlation matrix of inverted parameters from 50 GA runs of Cu^SiN^ membrane

|  | *C*_11_ | *C*_12_ | *C*_13_ | *C*_33_ | *C*_44_ | Thickness |
| --- | --- | --- | --- | --- | --- | --- |
| *C*_11_ | **1** | **0.301464** | **0.619948** | **-0.30674** | **-0.41735** | **-0.35703** |
| *C*_12_ | **-** | **1** | **0.319423** | **-0.03327** | **-0.2614** | **-0.12014** |
| *C*_13_ | **-** | **-** | **1** | **-0.14448** | **-0.32694** | **-0.18426** |
| *C*_33_ | **-** | **-** | **-** | **1** | **0.697371** | **0.93841** |
| *C*_44_ | **-** | **-** | **-** | **-** | **1** | **0.882664** |
| Thickness | **-** | **-** | **-** | **-** | **-** | **1** |

**Table S7** Statistical summary of inverted parameters of Cu^Si^ membrane

| Parameter | Mean±Std (GPa/nm) |
| --- | --- |
| *C*_11_ | **166.74 ± 7.51** |
| *C*_12_ | **79.22 ± 9.01** |
| *C*_13_ | **74.06 ± 9.32** |
| *C*_33_ | **189.92 ± 5.81** |
| *C*_44_ | **133.58 ± 4.65** |
| Thickness | **623.12 ± 8.61 nm** |

**Table S8** Correlation matrix of inverted parameters from 50 GA runs of Cu^Si^ membrane

|  | *C*_11_ | *C*_12_ | *C*_13_ | *C*_33_ | *C*_44_ | Thickness |
| --- | --- | --- | --- | --- | --- | --- |
| *C*_11_ | **1** | **-0.18191** | **0.479166** | **-0.19995** | **-0.29758** | **-0.18995** |
| *C*_12_ | **-** | **1** | **-0.13315** | **0.099949** | **0.069868** | **0.045124** |
| *C*_13_ | **-** | **-** | **1** | **-0.18317** | **0.421399** | **-0.15657** |
| *C*_33_ | **-** | **-** |  | **1** | **0.264889** | **0.965054** |
| *C*_44_ | **-** | **-** | **-** | **-** | **1** | **0.318524** |
| Thickness | **-** | **-** | **-** | **-** | **-** | **1** |

**5.2 Sensitivity Analysis**

To visualize the topology of the objective function and quantitatively evaluate parameter coupling as well as potential error propagation, one-dimensional fitness landscapes (**Figures S15 and S16**) and two-dimensional slices of the multi-objective fitness function (**Figures S17 and S18**) are computed around the optimal solution obtained from the 50 independent GA runs. For the 1D landscapes, each panel shows the fitness value as a function of one parameter while the other five parameters are fixed at their ensemble-averaged optimal values. For the 2D landscapes, each panel shows a slice for a pair of parameters while the remaining four parameters are fixed at their averaged optimal values.

The results reveal clear differences in parameter resolvability. For both membranes, *C*₃₃, *C*₄₄, and membrane thickness *h* are particularly sensitive, exhibiting sharp, prominent single peaks in the 1D fitness curves with relatively large fitness variations on the vertical axis (Δfitness ≈ 0.01–0.04). Specifically, *C*₃₃ peaks at approximately 177 GPa for Cu^SiN^ and 190 GPa for Cu^Si^, *C*₄₄ peaks at approximately 44 GPa for Cu^SiN^ and 140 GPa for Cu^Si^, and *h* peaks at approximately 530 nm for Cu^SiN^ and 620 nm for Cu^Si^. The corresponding 2D slices also display well-defined, localized global minima. *C*₁₁ and *C*₁₃ demonstrate moderate sensitivity, with relatively small variations in fitness value on the vertical axis (Δfitness ≈ 0.002–0.004) in the 1D landscapes (*C*₁₁ around 175 GPa for Cu^SiN^ and 160 GPa for Cu^Si^; *C*₁₃ around 92 GPa for Cu^SiN^ and 78 GPa for Cu^Si^) and reasonably distinct but less pronounced minima in the 2D contours, indicating that these parameters can still be determined with acceptable reliability. In contrast, the fitness landscapes involving *C*₁₂ are essentially flat in both 1D and 2D analyses, which shows almost no variation with changes in *C*₁₂. This near-complete insensitivity arises because, in transversely isotropic materials with out-of-plane detection and SH-mode suppression, *C*₁₂ is largely decoupled from the observable Lamb-wave dispersion.

Overall, the sensitivity analysis confirms that the SAFE-GA inversion can robustly recover *C*₃₃, *C*₄₄, and thickness *h* with high sensitivity, *C*₁₁ and *C*₁₃ with moderate sensitivity, while *C*₁₂ remains difficult to determine accurately due to its weak coupling with the detected wavefield. These results provide strong visual evidence for the stability and reliability of the inversion framework under the current experimental configuration.

|   **(a)** |   **(b)** |
| --- | --- |
|   **(c)** |   **(d)** |
|   **(e)** |   **(f)** |

**Figure S15** 1D Sensitivity analysis for Cu^SiN^ membranes. Each panel shows the fitness value as a function of one parameter while the other five parameters are fixed at their ensemble-averaged optimal values from 50 independent GA runs. The red star marks the location of the maximum fitness in each 1D landscape.

|   **(a)** |   **(b)** |
| --- | --- |
|   **(c)** |   **(d)** |
|   **(e)** |   **(f)** |

**Figure S16** 1D Sensitivity analysis for Cu^Si^ membranes. Each panel shows the fitness value as a function of one parameter while the other five parameters are fixed at their ensemble-averaged optimal values from 50 independent GA runs. The red star marks the location of the maximum fitness in each 1D landscape.

|   **(a)** |   **(b)** |
| --- | --- |
|   **(c)** |   **(d)** |
|   **(e)** |   **(f)** |

**Figure S17** 2D Sensitivity analysis for Cu^SiN^ membranes. Each panel shows a slice of the multi-objective fitness landscape for a pair of parameters while the remaining four parameters are fixed at their ensemble-averaged optimal values from 50 GA runs. The color scale represents the negative fitness value (lower values indicate better agreement with the experimental k–f map). Black contour lines highlight iso-fitness levels, and the red star marks the unique global minimum in each slice.

|   **(a)** |   **(b)** |
| --- | --- |
|   **(c)** |   **(d)** |
|   **(e)** |   **(f)** |

**Figure S18** 2D Sensitivity analysis for Cu^Si^ membranes. Each panel shows a slice of the multi-objective fitness landscape for a pair of parameters while the remaining four parameters are fixed at their ensemble-averaged optimal values from 50 GA runs. The color scale represents the negative fitness value (lower values indicate better agreement with the experimental k–f map). Black contour lines highlight iso-fitness levels, and the red star marks the unique global minimum in each slice.

**References**

1 Kammuri, K.; Miki, A.; Takeuchi, H. Reliable Young’s Modulus Value of High Flexible, Treated Rolled Copper Foils Measured by Resonance Method. Journal of Microelectronics and Electronic Packaging 2017, 14 (2), 70–76.
2 Kaneko, H.; Eguchi, T.; Inoue, H. Crystal Orientation Distribution and Elastic Anisotropy in Cu–Ni–Si Alloy Sheets. Furukawa Review 2016, 47, 40–43.
3 He, Y.; Luo, G.; Huang, J.; Li, Y.; Sohn, H.; Su, Z. Ultrafast laser-enabled optoacoustic characterization of three-dimensional, nanoscopic interior features of microchips. Ultrasonics 2025, 146, 107510.
